# Identifying Human PTP1B Enzyme Inhibitors from Marine Natural Products: Perspectives for Developing of Novel Insulin-Mimetic Drugs

**DOI:** 10.3390/ph15030325

**Published:** 2022-03-08

**Authors:** Marcello Casertano, Massimo Genovese, Lucia Piazza, Francesco Balestri, Antonella Del Corso, Alessio Vito, Paolo Paoli, Alice Santi, Concetta Imperatore, Marialuisa Menna

**Affiliations:** 1 Department of Pharmacy, University of Naples “Federico II”, 80131 Naples, Italy; marcello.casertano@unina.it (M.C.); alessio.vito@unina.it (A.V.); cimperat@unina.it (C.I.); 2 Department of Experimental and Clinical Biomedical Sciences, University of Florence, 50134 Florence, Italy; m.genovese13@gmail.com (M.G.); alice.santi@unifi.it (A.S.); 3 Biochemistry Unit, Department of Biology, University of Pisa, 56123 Pisa, Italy; l.piazza@studenti.unipi.it (L.P.); francesco.balestri@unipi.it (F.B.); antonella.delcorso@unipi.it (A.D.C.)

**Keywords:** marine natural products, marine-inspired chemical libraries, protein tyrosine phosphatase 1B, insulin-mimetic drugs, aldose reductase, type 2 diabetes mellitus

## Abstract

Diabetes mellitus (DM) represents a complex and multifactorial disease that causes metabolic disorders with acute and long-term serious complications. The onset of DM, with over 90% of cases of diabetes classified as type 2, implies several metabolic dysfunctions leading to consider DM a worldwide health problem. In this frame, protein tyrosine phosphatase 1B (PTP1B) and aldose reductase (AR) are two emerging targets involved in the development of type 2 diabetes mellitus (T2DM) and its chronic complications. Herein, we employed a marine-derived dual type inhibitor of these enzymes, phosphoeleganin, as chemical starting point to perform a fragment-based process in search for new inhibitors. Phosphoeleganin was both disassembled by its oxidative cleavage and used as model structure for the synthesis of a small library of functionalized derivatives as rationally designed analogues. Pharmacological screening supported by in silico docking analysis outlined the mechanism of action against PTP1B exerted by a phosphorylated fragment and a synthetic simplified analogue, which represent the most potent inhibitors in the library.

## 1. Introduction

In addition to the continued progress toward understanding the key cellular and molecular processes involved in the pathobiology of a given disease and, thus, validating suitable drug targets, the major problem in drug discovery remains the availability of novel chemical scaffolds. Large chemical libraries with associated high chemical diversity are needed to identify new lead compounds; in the current genomic era, in which tens of thousands of potential drug targets have been identified, the challenge of success actually lies in the number of small molecules that can be used as their modulators. Large and focused molecules libraries can be obtained by combinatorial chemistry approaches, in which chemical structures generated by computer software are prepared through systematic and repetitive linkage of various “building blocks”. An advantageous alternative to combinatorial chemistry as a resource of chemical diversity are Natural Products (NPs), which offer particularly favourable features for drug discovery, first their huge scaffold diversity and structure complexity. Unsurprisingly, it has been estimated that more than 65% of clinical drugs were derived from nature or they have been designed employing NP scaffolds as inspiring tools [1,2].

In recent years, there has been growing interest in Marine Natural Products (MNPs), which are often structurally very different from terrestrial ones, with unusual scaffolds and functional groups, and with which is associated a high potential for the discovery of new leads with particular mechanisms of action [2,3,4]. The main limitation of MNPs is that they are usually isolated in low amounts, sufficient for elucidation of their structure but inadequate for extensive drug screening [5]. However, it should be considered that unaltered NPs generally possess suboptimal pharmacological properties and, therefore, strategies to obtain more active, better absorbed, and less toxic analogues are almost always required to design valuable new drugs. In this scenario, organic synthesis proves to be a powerful tool to enhance the potential of MNPs; total chemical synthesis of MNPs, semi-synthesis using MNPs as a starting point, and synthesis of MNPs analogues, are some of the feasible strategies to enrich screening libraries with an adequate number of MNPs and MNP-inspired molecules. “Disassembling” the structure of an MNP model is indeed an innovative and effective strategy to investigate the structural requirements for its pharmacological effects and structure-activity relationships (SARs). This approach takes the form of a sort of *fragment-based screening* in which a fragments library inspired to the parent bioactive NP is produced through the design of a versatile and efficient synthetic protocol [6,7]. Fragments can be generally defined as poorly functionalized molecules with significantly reduced molecular weights; this corresponds to a lower structural complexity and, thus, to a more synthetic accessibility. The fragments are likely to exhibit lower affinity for biological targets than natural models, but this event is ultimately not a problem as these scaffolds can be appropriately manipulated/decorated for further optimization, thereby increasing the interaction with selected enzymatic counterpart(s) [8,9].

**Figure 1:**
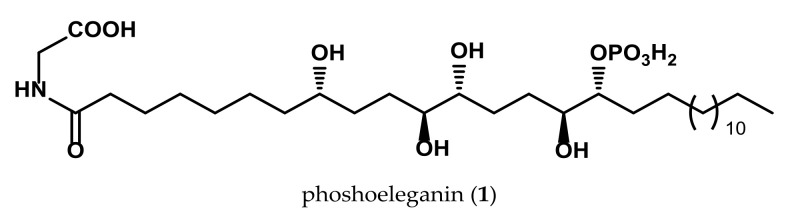
Structure of phosphoeleganin (**1**).

We have recently discovered phosphoeleganin (**1**) (Figure 1), a marine derived phosphorylated polyketide, as a dual inhibitor of the Protein Tyrosine Phosphatase 1B (PTP1B) and Aldose Reductase (AR) enzymes [10,11,12]. Both PTP1B and AR are considered as emerging targets involved in the onset and progression of chronic multifactorial diseases, such as type 2 Diabetes Mellitus (T2DM), obesity, cancer and, more generally, inflammatory-based diseases [13,14]. Phosphoeleganin has been demonstrated to inhibit both enzymes, acting, respectively, as a pure non-competitive inhibitor of PTP1B and a mixed-type inhibitor of AR. In addition, tests carried out using HepG2 cells confirmed that phosphoeleganin possesses an insulin-sensitizing activity [12]. To further explore and optimize the potential of this MNP as antidiabetic lead, as well as to overcome the supply issue, a common hurdle in the NPs based drug discovery, we have chemically manipulated the natural metabolite. In addition, we have synthesized a set of its small-size and less complex fragments by gradually disassembling the whole structure skeleton of the parent molecule. Herein, we describe the synthesis and the pharmacological characterization of the obtained fragments library as PTP1B and AR inhibitors.

## 2. Results

### 2.1. Oxidative Cleavage of Phosphoeleganin Affords Compounds ***2*** and ***3***

**Scheme 1:**
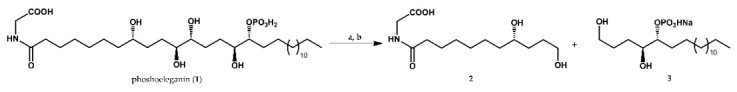
Synthesis of compounds **2** and **3** by cleavage of **1.** Reagents and conditions: (**a**) NaIO_4_, MeOH, rt, 3h. (**b**) NaBH_4_, 0 °C, 1.5 h.

A sample of phosphoeleganin (**1**), (10 mg), available at Department of Pharmacy of University of Naples Federico II was subjected to an oxidative cleavage as previously reported [10] to obtain compounds **2** and **3** in pure state (Scheme 1) [10].

### 2.2. Synthetic Approach for Obtaining Compounds ***4a**–**9b***

**Scheme 2:**
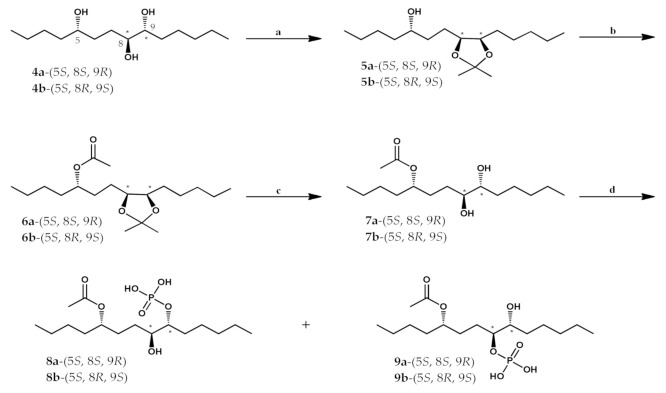
Reagents and conditions: (**a**) 2,2-dimethoxypropane, *p*-toluenesulfonic acid, rt, overnight. (**b**) Ac_2_O, pyridine, rt, overnight. (**c**) HCl 1%, MeOH/H_2_O 9:1, rt, overnight. (**d**) (1) Cl_3_CCN; (2) (*n*-Bu)_4_NH_2_PO_4_, CH_3_CN, rt, 2h. The symbol * represents relative configuration.

We had previously synthesized the enantiomerically pure diastereoisomeric model structures **4a** and **4b**, which are endowed with absolute configuration 5*S*, 8*S*, 9*R* and 5*S*, 8*R*, 9*S*, respectively, to complete the stereostructural assignment of phosphoeleganin (**1**) [11]. Starting by triols **4a** and **4b**, we have prepared the small molecules **4a**–**9b** through a series of simple and highly efficient reactions sequence (Scheme 2). Firstly, the triols **4a** and **4b** were separately dissolved in 2,2-dimethoxypropane with a catalytic amount of *p*-toluenesulfonic acid by keeping the mixture under magnetic stirring overnight and by introducing a protective group on the 1,2-diol system at C8-C9. The alkaline quenching (aqueous saturated solution of NaHCO_3_) and the solvent removal in vacuo afforded the acetonide intermediates **5a** and **5b** sufficiently pure for the subsequent step. Indeed, compounds **5a** and **5b** were directly treated with acetic anhydride in pyridine by stirring the mixture at rt overnight. Then, the mixture was cooled and subjected to addition of an excess of MeOH to make faster the solvent removal in vacuo. The introduction of the acetyl group at C5 on the starting compounds was efficient since it afforded compound **6a** and **6b** in high yield and pure form. Accordingly, the two compounds directly underwent the hydrolysis of acetonide functionality at C8-C9 by using acidic condition (1% HCl) which allowed to afford the acetylated intermediates **7a** and **7b** in excellent yield.

The acetylated derivatives **7a** and **7b** were then converted into the relevant two phosphate monoesters by a one-pot method of phosphorylation reaction. This synthesis is in-depth performed as the introduction of a phosphate group on a carbon skeleton can widely modify the pharmacokinetic and pharmacodynamic properties of a molecular scaffold. Moreover, several hindrances (e.g., the formation of pyrophosphate products as side compounds and/or the need to use a large excess either of the substrate or the phosphorylation reagent) were described within the previously proposed protocols [15]. Lira and co-workers proposed an improved one-pot method that could increase the yield of phosphate monoesters and avoid the production of pyrophosphates [16]. The straightforward method starts from the commercially available tetrabutylammonium dihydrogen phosphate ((*n*-Bu)_4_NH_2_PO_4_) which reacts with trichloroacetonitrile giving rise to a mixed anhydride-like activated phosphate, i.e., the trichloroacetimidate. The latter is subjected to nucleophilic attack by the hydroxyl group of the alcohol yielding the corresponding phosphate monoester and trichloroacetamide [16].

Compounds **7a** and **7b** were separately dissolved in dry CH_3_CN whereas trichloroacetonitrile was added to mixture. Subsequently, phosphate salt solubilized in CH_3_CN was slowly added and the resultant mixture stirred for 2 h at rt (Scheme 2). After solvent removal under N_2_, each raw material was firstly chromatographed by HPLC on reversed phase (MeOH/H_2_O 75:25 + 0.1% TFA as mobile phase) collecting a fraction composed of a mixture of **8a/9a** and **8b/9b**, respectively. Thus, the different phosphorylated derivatives **8a** and **9a** as well as **8b** and **9b** were obtained as individual and pure compounds by a further HPLC on reversed phase (eluting with MeOH/H_2_O 6:4 + 0.1% TFA).

All compounds have been completely characterized by NMR spectroscopy and HRESI mass spectrometry to unequivocally define the whole structure and to distinguish among the structural isomers. The ^1^H and ^13^C NMR chemical shifts of the phosphorylated compounds **8a**–**9b** are reported in Table 1.

### 2.3. Preliminary Screening on AR and PTP1B Enzymes of Compounds ***2**–**4b*** and ***7a**–**9b***

To assess the effects of compounds **2**–**4b** and **7a**–**9b** on catalytic activity of both AR and PTP1B, we performed a preliminary enzymatic screening assay using a fixed concentration (20 µM) of each compound. In the case of AR, the residual activity measured was above 95% for all the tested compounds. For some of them it was possible to increase their concentration in the assay without any interference in the spectrophotometric measurements. However, the observed inhibitory effect was never greater than 15%, except for **8b** and **9b**, for which a residual activity of 76 and 77%, respectively, was observed at concentrations of 210 µM (Table S1, Supplementary Materials).

**Figure 2:**
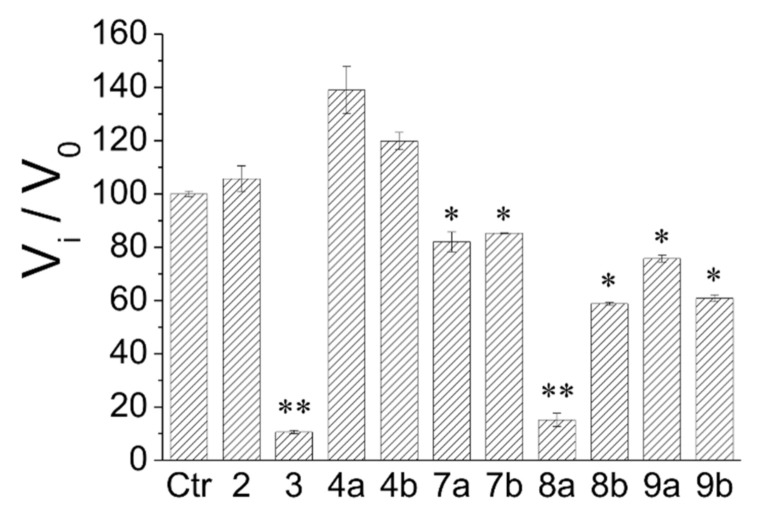
Preliminary screening assay on PTP1B. All assays were started by diluting an aliquot of PTP1B in the assay solution containing substrate alone (control test) or substrate and an aliquot of each compound (20 µM, final concentration). After 30 min, the reactions were stopped by adding a concentrated KOH solution. The enzyme activity was determined by measuring the amount of p-nitrophenol that was released in each sample using a spectrophotometer. Data obtained were normalized respect to control sample. Data showed in the figure represent the mean value ± SD (*n* = 3). Statistical analysis was carried out using one way ANOVA followed by Tukey’s HSD test for pairwise comparisons. Statistics have been drawn on raw data and compare samples to control (Ctr). * *p* < 0.05; ** *p* < 0.01.

On the other hand, compounds **3**, **8a**, **8b, 9a**, and **9b** exerted an interesting inhibition potential by reducing PTP1B activity at the tested concentration (Figure 2).

It is interesting to note that all the PTP1B inhibiting compounds (**3**, **8a**, **8b**, **9a**, and **9b**) have a phosphate group, thus suggesting that this moiety has a role in the inhibition of enzyme activity. The semisynthetic compound **3**, resulting from a chemical manipulation of the bioactive parent compound **1** and most closely structurally related to it, is the most potent in the series; however, compound **8a** also showed significant inhibition power, and the added value of this compound is that it is a derivative easily obtained by synthesis. Moreover, comparison of the activity of **8a** with **8b** (with different stereochemistry at the phosphorylated diol system) and between **8a** and **9a** (with a different regiochemistry at the phosphorylated diol system), suggested that both position on the alkyl chain and stereochemistry of the phosphate moiety are key factors for the inhibitory power of these compounds.

**Figure 3:**
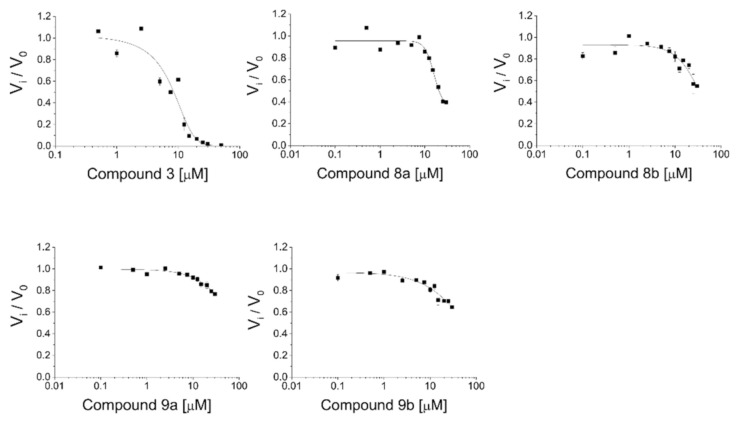
Determination of the IC_50_ values with compounds **3**, **8a**, **8b**, **9a** and **9b**. An aliquot of PTP1B enzyme was diluted in the presence of increasing concentration of the compounds. The activity of PTP1B was determined by measuring the absorbance of the sample at 400 nm, using a spectrophotometer. Data obtained were normalized respect to the control sample and analysed using a non-linear fitting software. Data showed in the figure represent the mean value ± SD (*n* = 3).

To better evaluate the inhibitory power of the most active compounds, we determined their IC_50_ values on PTP1B (Figure 3); the IC_50_ values are shown in Table 1, reporting also, as positive control, the IC_50_ value determined for phosphoeleganin (**1**).

As showed in Figure 3 and Table 1, our results confirmed compounds **3** and **8a** as the most active PTP1B inhibitors, with an IC_50_ value in the low-micromolar range. Interestingly, the IC_50_ values determined for phosphoeleganin (0.7 ± 0.1 µM) and **3** (6.7 ± 3.3 µM) were one order magnitude lower than the one previously determined (11 ± 2 and 24 ± 7 µM, respectively) [10]. The observed differences may be attributable to the different experimental conditions that have been used to perform the activity assays, such as a different pH value and the use of a GST–PTP1B fusion protein instead of purified PTP1B alone (1-302 aa).

### 2.4. Definition of ***3*** and ***8a*** Mechanism of Action on PTP1B

**Figure 4:**
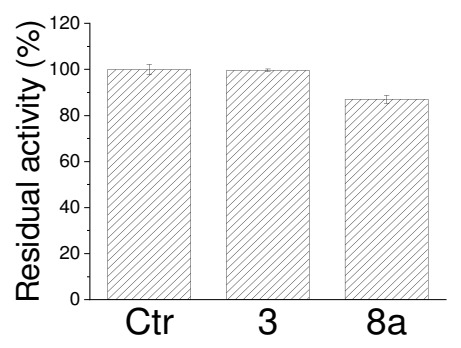
Dilution assay. To evaluate the mechanism of action of the compounds, a reversibility assay was carried out. The PTP1B was incubated in the presence of the saturating concentration of each compound (50 µM) for 1 h at 37 °C. Then, an aliquot of enzyme was diluted 500 folds in the assay buffer to evaluate the residual activity of the enzyme. Tests were carried out in triplicate. Data represent the mean value ± SD.

To better understand the mechanism of action of **3** and **8a**, we performed a dilution assay test to evaluate whether these compounds behave as reversible or non-reversible inhibitors. We observed that after incubation of PTP1B with both compounds, the enzyme recovered its activity almost completely upon dilution in the assay buffer. This finding suggests that such compounds behave as reversible inhibitors of PTP1B (Figure 4).

**Figure 5:**
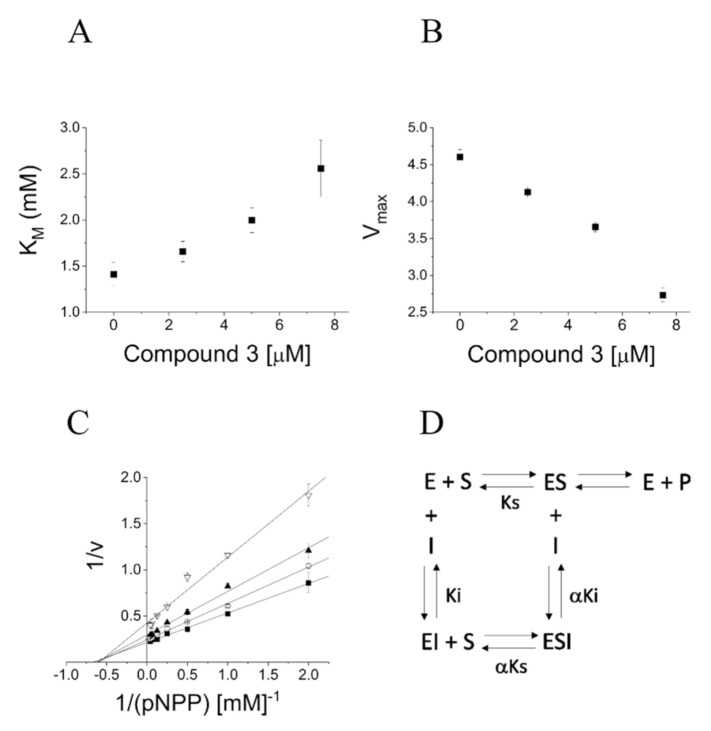
Kinetic characterization of **3** as PTP1B inhibitor. The concentrations of compound **3** used were 0 (■), 2.5 (○), 5 (▲) and 7.5 (▽). Dependence of main kinetic parameters K_M_ (**A**) and V_max_ (**B**) from the concentration of compound **3**. Lineweaver-Burk plot (**C**). Bars represent the standard deviations of the means from at least three independent measurements (if not present, they are within the symbol’s size). Panel **D** shows the mechanism of action of a linear mixed type inhibitor. E, free enzyme; S, substrate; ES, enzyme-substrate complex; EI, enzyme-inhibitor complex; ESI the enzyme-substrate-inhibitory ternary complex; Ks, dissociation constant of the enzyme-substrate complex; Ki, dissociation constant of EI complex; αKs and αKi, dissociation constant of enzyme-substrate-inhibitor complex (α > 1).

Furthermore, to elucidate the inhibitory mechanism of **3** on PTP1B, we studied the dependency of K_M_ and V_max_ on its concentration (Figure 5A,B). We observed that the increase of inhibitor concentration was accompanied by a decrease of V_max_ value and an increase of K_M_. Furthermore, Lineweaver-Burk plot (Figure 5C) shows that experimental points described straight lines that intersect one each other in the left panel; taken together, these data suggested that compound **3** behaved as a linear mixed-type inhibitor (Figure 5D).

**Figure 6:**
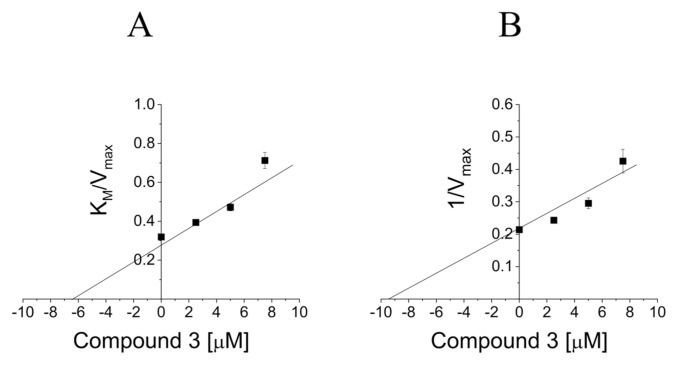
Secondary plots. K_M_/V_max_ (**A**) and 1/V_max_ (**B**) versus the inhibitor concentration, respectively. The intercept of straight line with the “x” axis in Panel A, represents the Ki value, while the intercept of straight line with the “x” axis in Panel B represents the Ki′ value (Ki′ = αKi). The symbols ■ represent respectively the slopes (**A**) and the intercepts (**B**) of the straight lines shown in Figure 5C.

Finally, by analysing the behaviour of K_M_/V_max_ (Figure 6A) and 1/V_max_ (Figure 6B) with respect to the inhibitor concentration, we calculated both the values of Ki (6.6 ± 0.8 µM), Ki′ (9.4 ± 0.6 µM) and of α (1.4 ± 0.2).

**Figure 7:**
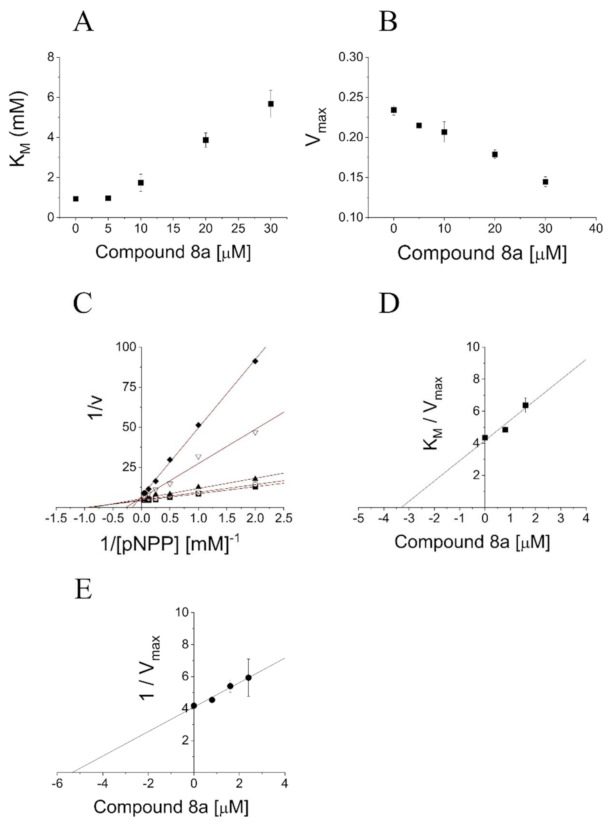
Kinetic characterization of **8a** as PTP1B inhibitor. The concentrations of compound **3** used were 0 (■), 5 (○), 10 (▲), 20 (▽) and 30 (♦) µM. Dependence of K_M_ (**A**) and V_max_ (**B**) from the inhibitor concentration. Lineweaver-Burk graph (**C**). Bars represent the standard deviations of the means from at least three independent measurements (if not present, they are within the symbol’s size). Secondary plots: K_M_/V_max_ (**D**) and 1/V_max_ (**E**) versus the inhibitor concentration, respectively. The intercept of straight line with the “x” axis in Figure D represents the Ki value, while the intercept of straight line with the “x” axis in Figure E represents the Ki′ value (Ki′ = αKi).

**Figure 8:**
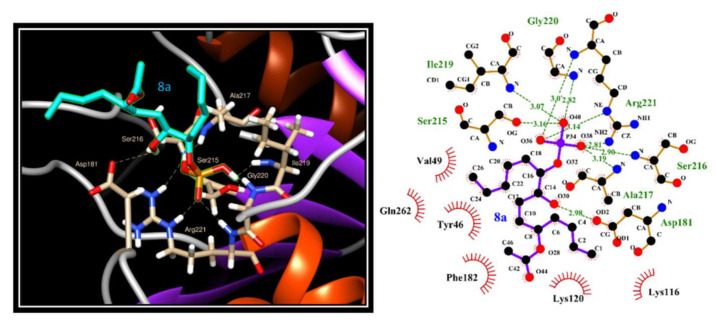
Visualization of the interaction between compound **8a** and the active site of PTP1B.

A similar analysis was carried out with compound **8a** (Figure 7). As reported in Figure 7, incubation of PTP1B with **8a** resulted in an increase in K_M_ and a decrease in V_max_, suggesting that it behaved as a mixed type inhibitor. According with this hypothesis, the Lineweaver-Burk plot describes straight lines that intersect one to each other in a point in the left side of the graphic. Therefore, using an approach similar to that above described, we calculated the values of K_i_ (3.3 ± 0.4 µM), Figure 8D, of K_i_’ (5.4 ± 0.2 µM) and α (1.6 ± 0.1), Figure 7E. Overall, these results suggest that phosphoeleganin and **8a** have a similar kinetic behaviour.

### 2.5. Evaluation of **3** and **8a** Interaction with PTP1B by In Silico Docking Analyses

The interaction mode of **3** and **8a** with PTP1B was determined by performing an in silico docking analysis, using the AutoDock software. We found that the most favorable position localized **8a** into the active site of the enzyme (Figure 8). In this pose, the phosphate moiety of **8a** made several hydrogen-bonds with the side chain of Arg221 and with some nitrogen atoms of the amino acids belonging to *p*-loop.

**Figure 9:**
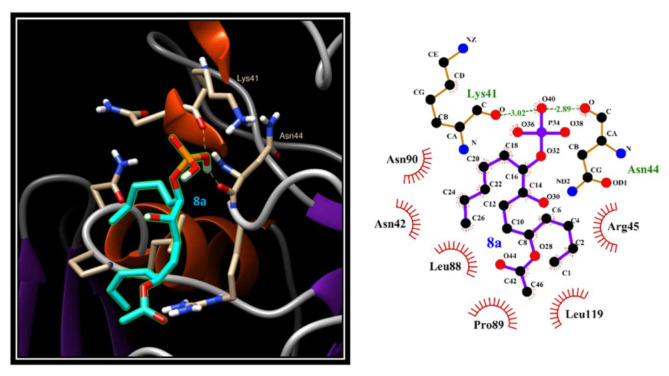
Interaction of compound **8a** with the region lining the active site of PTP1B enzyme.

Moreover, one hydroxyl group of **8a** engaged a hydrogen bond with the side chain of Asp181, the essential catalytic residue belonging to WPD loop [17]. Of note, in the pose 2, the phosphate moiety of **8a** occupied a distinct area that involved the Lys41 and Asn44 residues, while the aliphatic chain engaged extensive hydrophobic interaction with Asn42, Arg45, Leu88, Pro89, Asn90, and Leu119 residues (Figure 9).

Several of these residues have been previously involved in the formation of the second aryl phosphate binding site located near the active site [18]. This evidence supports the kinetic data, which showed that compound **8a** behaved as a mixed-type non-competitive inhibitor.

**Figure 10:**
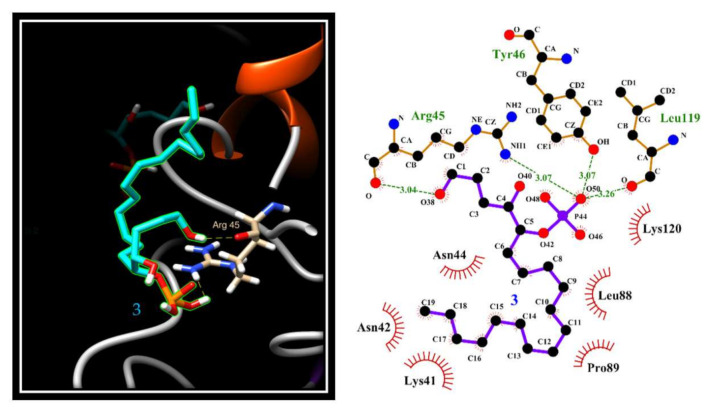
Interaction of compound **3** with the region lining the active site of PTP1B.

On the other hand, the results of docking analyses carried out with compound **3** revealed multiple interaction modes of this molecule with PTP1B. The most energetically favourable pose localized **3** near the active site (Figure 10). 

**Figure 11:**
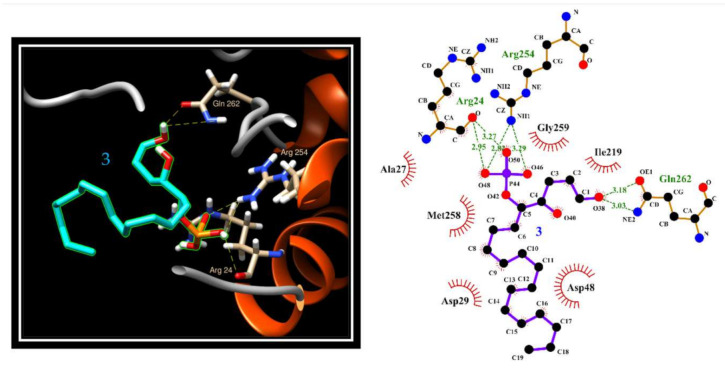
Interaction of compound **3** within the second aryl binding site of PTP1B enzyme.

In this case, the interactions of the phosphate group of **3** with Arg45 and Tyr46 side chain and with oxygen atom of carbonyl group of Leu119, contributed to firmly bind the inhibitor on PTP1B surface. Considering that Tyr46 plays a role in stabilizing the interaction of the substrate with the active site of the enzyme [19], we have assumed that the binding of 3 in this position is able to hinder the enzyme-substrate interaction. Furthermore, the result of docking analysis revealed that compound **3** can also interact with residues that form the p-loop. We observed that oxygen atoms of phosphate moiety form hydrogen bonds with the amino groups of the Arg254 residue (Figure 11). In the same moment, the -OH group of **3** engaged hydrogen bonds with the side chain of Gln262, which is an essential residue for the catalysis.

### 2.6. Analysis of Insulin Signalling Pathway in C2C12 Cells Treated with Compound ***3***

**Figure 12:**
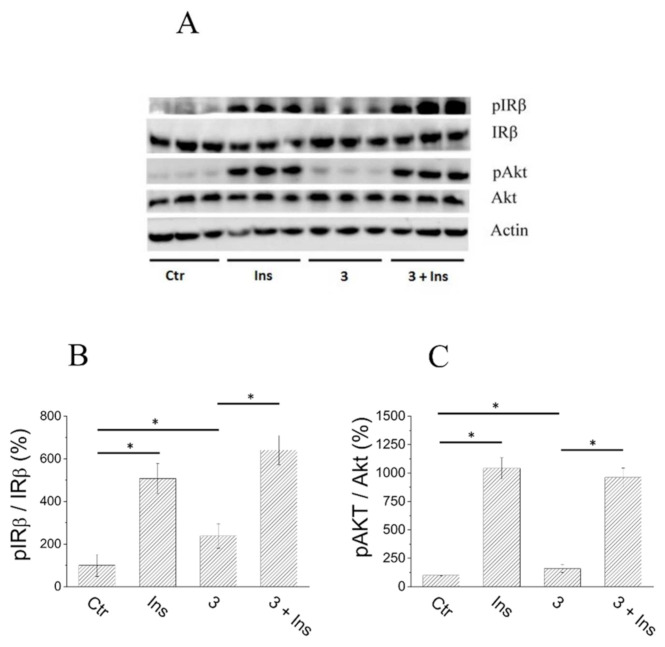
Effect of compound **3** on insulin signalling pathway. C2C12 cells were incubated with compound **3** (20 µM final concentration) for 30 min, in the presence or not of insulin (10 nM). After that, cells were lysed, and cellular extracts were analysed to evaluate the phosphorylation status of IR and Akt. (**A**), western blot image; (**B**,**C**), quantitation of western blot carried out using Kodak MI software. Data were normalized respect to control sample. All tests were carried out in triplicate. Ctr: control experiment; Ins: cells treated with 10 nM insulin; **3**: cells treated with compound **3** (20 µM); **3** + Ins, cells treated with insulin and compound **3**. Statistical analysis was carried out using one way ANOVA followed by Tukey’s HSD test for pairwise comparisons. * *p* < 0.05.

The ability of compound **3** and **8a** to improve the insulin signalling pathway was evaluated in murine myoblast C2C12 cells. We found that the treatment of C2C12 cells with compound **8a** did not improve the insulin signalling pathway (data not shown), while phosphorylated fragment **3** did it (Figure 12). For this reason, we focused our attention on compound **3**. We treated differentiated C2C12 cells with 20 µM of compound **3**, in the presence or not of insulin. We found that in C2C12 cells treated with **3** alone, the phosphorylation levels of both insulin receptor (IR) and its downstream kinase Akt increased with respect to control cells, suggesting that **3** acts as an insulin-mimetic agent. On the other hand, IR and Akt phosphorylation levels in C2C12 cells that were treated with insulin alone were not statistically different compared to the combined treatment of insulin with **3**. Taken together, these results demonstrated that **3** acts as an insulin-mimetic agent but its effects on insulin signalling pathway are probably hidden when insulin is present.

### 2.7. Glucose Uptake Assay of Compound ***3***

**Figure 13:**
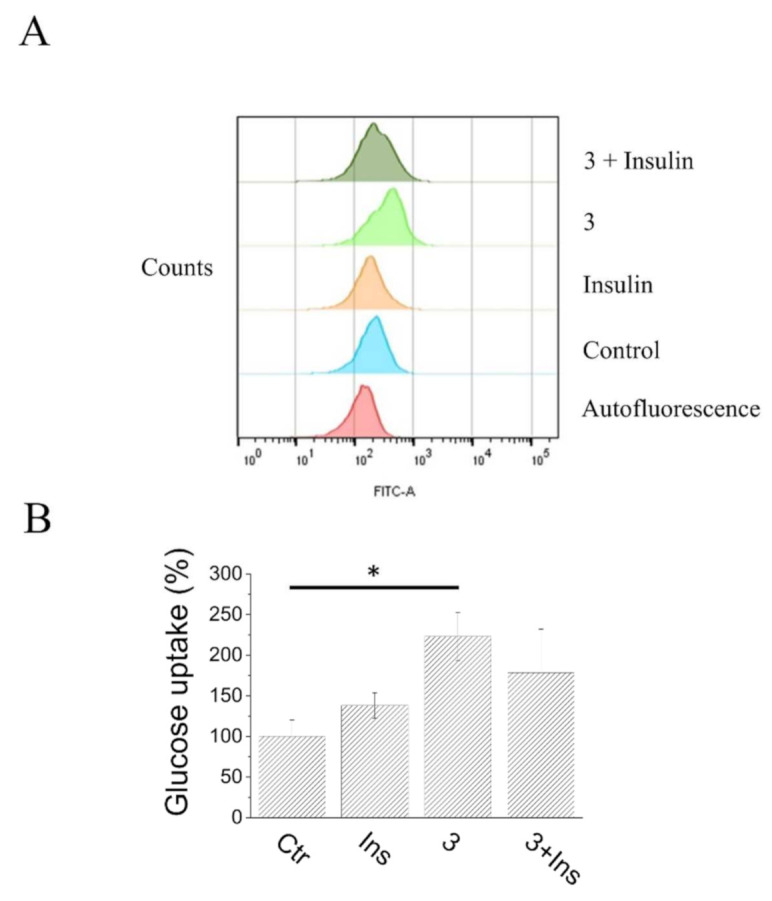
Glucose uptake assay. C2C12 cells were treated with 10 nM insulin, 20 µM compound **3** or **3**-insulin combination and then incubated in the presence of 2-NBDG for 3 h. After that, the glucose uptake was analysed by flow cytometry analysis. Each test was carried out in quadruplicate. For each test, 10,000 events at least were analysed. (**A**), quantification of the mean fluorescence values of C2C12 cells. Data represents the mean value ± SD (*n* = 4). Statistical analysis was carried out using one way ANOVA followed by Tukey’s HSD test for pairwise comparisons. * *p*< 0.05. (**B**), representative cell fluorescence distribution obtained by analysing data with the FlowJo software. Red, cells autofluorescence; light blue, control cells; orange, C2C12 cells stimulated with 10 nM insulin; green, C2C12 cells stimulated with 20 µM **3**; dark green, C2C12 cells stimulated with **3**-insulin combination.

To further investigate the pharmacological behaviour of compound **3**, its ability to improve glucose uptake was evaluated using C2C12 cells. Interestingly, cells treated with **3** alone incorporated more glucose than insulin-stimulated C2C12 cells, while cells stimulated with the combination of **3** and insulin showed intracellular glucose levels similar to those found in the cells treated with the hormone alone (Figure 13). This finding reinforces the hypothesis that **3** acts as an insulin-mimetic compound.

## 3. Discussion

In a previous study, we have demonstrated that phosphoeleganin (**1**), a phosphorylated poliketide isolated from the Mediterranean ascidian *Sidnyum elegans*, is able to inhibit both AR and PTP1B, acting as an insulin-sensitizing agent [12]. Therefore, in order to develop compound **1** as antidiabetic lead, we have carried out a sort of fragment-based approach by chemical manipulation of the natural metabolite and by synthesis of its simplified analogues. The guideline behind this approach has been mainly the need to overcome the supply issue, a common hurdle in the NPs based drug discovery. Thus, we have decided to obtain/synthesize small fragments inspired to the most functionalized part of the parent bioactive NP by an easy and inexpensive way, even in terms of atom economy. Therefore, we firstly performed the easily achievable oxidative cleavage of the 1,2-diol system of 1, affording the two fragments, **2** and **3** (Scheme 1), to simplify our synthetic targets as much as possible; we already knew, indeed, that the phosphate-containing fragment (compound **3**) maintains almost completely the activity on PTP1B [10]. Moreover, we have designed and synthesized a small chemical library of molecules, based on the structure of tetradecane-5,8,9-triol, inspired to the most functionalized part of the natural **1** (and, thus, to fragment **3**). Both semi-synthetic and synthetic molecules have been subjected to pharmacological evaluation to assess their PTP1B and AR enzymes inhibitory activity.

For the production of the chemical library, we have used as starting material the (5*S*,8*S*,9*R*)-tetradecane-5,8,9-triol (**4a**) and (5*S*,8*R*,9*S*)-tetradecane-5,8,9-triol (**4b**) diastereoisomers. Compounds **4a** and **4b** have been prepared in the enantiomerically pure form, according to our previously reported synthetic procedure [11]. The whole adopted synthetic protocol (Scheme 2) was based on the use of commercially available reagents with different low-cost steps and concerned sequentially protection and deprotection reactions. Interestingly, the conversion of intermediates **7a** and **7b** into the corresponding monophosphates **8a**/**9a** and **8b**/**9b**, was performed through a one-pot method whose advantages are the use of a readily available phosphate group donor, (*n*-Bu)_4_NH_2_PO_4_, and an increased yield of the monophosphate ester avoiding the formation of side pyrophosphate byproducts [16]. In addition, one-pot phosphorylation of compounds **7a** and **7b** allowed to selectively introduce a monophosphate ester functionality on their 1,2-diol system. Each compound was structurally characterized by its spectroscopic means (^1^H, ^13^C and HR-ESIMS); for this purpose, the analysis of the key correlations recorded by 2D NMR experiments (mainly ^1^H-^13^C-HSQC and ^1^H-^13^C-HMBC) guaranteed to define the structure of the synthesized fragment-type compounds, and in particular, to univocally and unequivocally distinguish from the structural isomers **8a**/**9a** and **8b**/**9b**.

Then, both phosphoeleganin fragments 2 and 3, as well as its synthetic simplified analogues 4a-9b were subjected to pharmacological screening to investigate the structural requirements underlying the inhibition of PTP1B and AR enzymes. The preliminary enzymatic screening on AR evidenced that the inhibitory effect showed by the natural metabolite phosphoeleganin (1) was completely lost in almost all the synthetic derivatives (a residual activity of 76 and 77 %, respectively, was observed at 210 µM only for compounds 8b and 9b). The semisynthetic fragments 2 and 3, obtained from the oxidative cleavage of 1, were ineffective in inhibiting AR enzyme, too, (Table S1). This finding can be interpreted in light of the previous results obtained from the in silico docking analysis of phosphoeleganin in the active site of AR [12]. The docking analysis indicated as relevant interactions for the inhibition of the catalytic activity of the enzyme those involving the more polar region of 1 (i.e., the region containing phosphate and OH groups). However, also the aliphatic portion of 1 (i.e., the C17-C30 region) contributed to the enzyme-ligand interaction, through the occurrence of several hydrophobic interactions at the level of the selectivity pocket, allowing the anchoring of the molecule. Moreover, the introduction of an acetyl group in some synthetic fragments, made to ensure the presence of a carbonyl group that is believed to be partially involved in AR inhibition, was not enough to save the inhibitory activity exerted by the whole molecule. Most likely, it is necessary to design fragments containing, in addition to the carbonyl and polar groups, an aliphatic portion of sufficient size to ensure their anchorage to the selectivity pocket.

The preliminary enzymatic screening on PTP1B revealed that compounds bearing a phosphate group (**3**, **8a**, **8b**, **9a** and **9b**) showed inhibitory activity and, among these, compounds **3** and **8a** were found the most active ones with an IC_50_ of 6.7 ± 3.3 µM and 16.0 ± 2.0 µM, respectively. Thus, the presence of a phosphate group seems to be essential for the inhibition of PTP1B; indeed, the absence of this chemical group causes loss of activity as observed for compounds **2**, **4a**/**4b**, and **7a**/**7b**. Moreover, a comparison of the activity of **8a** with **8b** (with different stereochemistry at the 1,2-monophosphorylated diol system) and between **8a** and **9a** (with a different regiochemistry at the 1,2-monophosphorylated diol system) suggested that both the position on the alkyl chain and stereochemistry of the phosphate moiety are key factors strongly affecting the inhibitory power of these compounds. It is noteworthy that the two most potent PTP1B inhibitors in the series, compounds **3** and **8a**, are featured by the same regiochemistry and absolute configuration of phosphoeleganin at the 1,2-monophosphorilated diol system. Nevertheless, the PTP1B inhibitory power of **3** and **8a** is an order of magnitude smaller than that of phosphoeleganin, at least, and this finding confirms the importance of the phosphoeleganin aliphatic chain in strengthening the interaction of the PTP1B inhibitor complex. It is important to highlight that, though less active than both the natural model compound **1** and compound **3**, deriving from chemical manipulation of **1**, compound **8a** can be easily, cheaply, and sustainably obtained by synthetic routes and, thus, it could represent an optimized hit to develop. In order to obtain as much information as possible about features and factors underlying the inhibition of PTP1B by phosphorylated derivatives, we have continued the in-depth pharmacological characterization on both compounds **3** and **8a**.

Kinetic analyses revealed that compound **3** and **8a** act as reversible, mixed-type non-competitive inhibitors, showing similar Ki values. These results are encouraging as a reversible and non-competitive PTP1B inhibition allows to reduce the toxicity caused by a prolonged and/or irreversible enzyme inhibition or by the inhibition of other enzymatic isoforms that are involved into different biological processes.

In silico docking analysis give important information about the interaction mode of compound **3** and **8a** with the PTP1B. We observed that the synthetic compound **8a** interacts with different regions of the enzyme’s surface. In the most favourable position, the phosphate of **8a** results firmly linked into the active site of PTP1B where it interacts with the side chain of Arg221, while the hydroxyl group located near the phosphate moiety makes a hydrogen bond with the side chain of Asp181, thus contributing to block the active site of the enzyme in the “closed conformation”. It is probable that the presence of phosphate group positioned in the middle of the aliphatic chain represents the main constrain that favours this interaction mode. Indeed, because of its hydrophobic properties, the aliphatic chain of **8a** cannot access to the active site zone, which results positively charged because of the presence of a protonated arginine residue side chain (Arg221). This constrain favours the interaction of the aliphatic chain of **8a** with some residues located in the slit of the active site (Tyr46, Ala49, Phe182) and the interaction of the hydroxyl group with Asp181, while the phosphate group can penetrate deeper into the active site of PTP1B.

In the second pose, the aliphatic chain of **8a** appears anchored in a hydrophobic task generated by the side chains of Asn42, Arg45, Leu88, Pro89, Asn90 and Leu119, while the phosphate group forms two hydrogen bonds with carbonyl group of Lys41 and Asn44 residue. Although located far from the active site, these residues are close to YRD motif (residues 46–48) whose correct positioning is determinant to maintain full catalytic activity of the enzyme [20]. Therefore, we have hypothesized that the interaction of **8a** in this region could distort the positioning of some essential residues resulting in an impairment of the catalytic process.

Compound **3** is structurally different from compound **8a** as it exhibits a longer side chain and a phosphate group positioned at the end of the molecule. Interestingly, the most favourable interaction sees compound **3** to interact with some amino acid located close the active site of PTP1B. In particular, the aliphatic chain of compound **3** interacts with the hydrophobic region generated by the side chains of Lys41, Asn42, Asn44, Leu88, Pro89 and Lys120, but its phosphate group moves deeper by making hydrogen bonds with the side chain of Arg45 and Tyr46. This last one residue is located in a surface loop between the α1 helix and the β1 strand and, together with Arg47 and Asp48, is directly involved in the substrate binding [21]. Moreover, in the second pose, phosphate moiety of compound **3** points to second aryl binding site where it makes hydrogen bonds with the carbonyl group of Arg24 and the nitrogen atoms of Arg254 side chain. In the same moment, terminal hydroxyl group of compound **3** forms hydrogen bonds with the side chain of the Gln262, a residue important for the positioning of the nucleophilic water molecule that contributes to the hydrolysis of the cysteinyl-phosphate intermediate during catalysis [22]. Overall, these results confirmed that both compounds **8a** and **3** can move inside the active site, or interact with different sites on the enzyme surface, confirming that they act as mixed-type non-competitive inhibitors.

The fragment **3** and the synthetic analogue **8a** were tested in C2C12 cells to investigate their impact on insulin signalling pathway. For what concern compound **8a**, it resulted completely inactive in C2C12 cells, whereas compound **3** showed an evident insulin-mimetic activity. Taking into account that both compounds behaved as good PTP1B inhibitors in vitro, to date it is not simple to justify their differential activity in C2C12 cells. Several causes could contribute to nullifying the activity of compound **8a** including poor bioavailability, its rapid degradation or the fact that, in the cellular environment, the interaction sites of compound **8a** on the surface of PTP1B are not accessible. Likewise, it is not easy to explain why compound **3** possesses insulin mimetic activity alone but is unable to increase insulin sensitivity when administered in combination with the hormone.

A similar problem has been addressed by other researchers engaged in the design and functional analysis of new small molecules as PTP1B inhibitors. John E. Bleasdale et al. showed that some cholecystokinin (CCK)-derived peptidomimetic compounds act as potent and specific inhibitors of PTP1B and promote IR activation. Indeed, they observed that in rat skeletal (L6) myoblast cells, one of these compounds increased the magnitude of tyrosine phosphorylation of IR and the rate of absorption of 2-[^3^H] deoxyglucose (2-DOG) even in the absence of insulin. This finding suggested that this molecule behaved such as an insulin-mimetic compound [23]. In 2003 Laiping Xie et al., studied the effect of new small molecules PTP1B inhibitors on insulin signalling pathway in different insulin-sensitive cell lines. They observed that the most potent molecules acted as both insulin mimetics and sensitizers, thereby validating the notion that small molecule PTP1B inhibitors could be used as antidiabetes drugs [24]. In 2004, Christian Wiesmann et al. reported that some benzbromarone derivatives behaved as potent and specific allosteric PTP1B inhibitors and were able to enhance insulin signalling in Chinese hamster ovary cells (CHO cells). Interestingly, the authors of this study demonstrated that such compounds not only acted as insulin sensitizing agents, but also were able to activate the insulin signalling pathway also in the absence of insulin co-stimulation. Based on these evidences, the authors concluded that these allosteric inhibitors also can act as insulin mimetic agents [17]. It is thus evident that the ability of PTP1B inhibitors to act as insulin-mimetic compounds is not a peculiarity of our compounds but rather a common property of other PTP1B inhibitors, as those above reported.

The insulin mimetic activity of PTP1B inhibitors could be explained by taking into account the peculiar structure of the insulin receptor. In fact, in contrast with most hormone or growth factor receptors, the insulin receptor is naturally dimeric and possesses intrinsic (constitutive) tyrosine kinase activity, even in the absence of insulin. Based on this evidence, it is reasonable to think that under starvation, the activity of PTP1B is necessary to dampen the spontaneous phosphorylation of the insulin receptor, thus contributing to its complete inactivation. Therefore, in this condition, inhibition of PTP1B activity would be expected to increase the basal level of IR phosphorylation, leading to the activation of insulin signalling.

## 4. Materials and Methods

### 4.1. Materials

Solvents, deuterated solvents and commercial reagents: Sigma-Aldrich (Saint Louis, MO, USA). TLC: Silica Gel 60 F254, plates 5 × 20, 0.25 mm, Merck (Kenilworth, NJ, USA). High-resolution MS (negative mode) was performed on a Thermo LTQ Orbitrap XL mass spectrometer (Thermo-Fisher, San Josè, CA, USA). The MS spectrum of PE was recorded by infusion into the ESI source using MeOH as solvent. ^1^H (700 MHz) and ^13^C (175 MHz) NMR were carried out on a Bruker Avance Neo spectrometer (Bruker BioSpin Corporation, Billerica, MA, USA); chemical shifts were referenced to the residual solvent signal (CD_3_OD: *δ*_H_ = 3.31, *δ*_C_ = 49.0) [25]. A Jasco P-2000 polarimeter (Jasco Europe s.r.l., Cremella, Italy) at the sodium D line was used to measure optical rotations. High performance liquid chromatography (HPLC) separation was achieved on a Knauer K-501 apparatus equipped with a Knauer K-2301 RI detector (LabService Analytica s.r.l., Anzola dell’Emilia, Italy).

For western blot analyses, the following antibodies were used: β-actin, clone C-4 (sc-47778) was from Santa Cruz Biotechnology (Santa Cruz, CA, USA); pAkt (9271S) and Akt (9272S) antibodies were from Cell Signalling Technology (Danvers, MA, USA). Secondary antibodies were from Santa Cruz Biotechnology (Santa Cruz, CA, USA).

L-idose and NADPH were from Carbosynth; *p*-nitrophenylphosphate (*p*-NPP) was from Chemcruz, Santa Cruz Biotechnology (Santa Cruz, CA, USA). All other chemicals were obtained from Merck Life Science s.r.l. (St. Louis, MO, USA), unless otherwise specified. Cell culture media and foetal bovine serum (FBS) were from Euroclone (Pero, Italy).

### 4.2. Synthesis of Compounds ***2*** and ***3***

Compounds **2** and **3** were obtained by carrying out the procedure that is reported in our previous work [10]. Phosphoeleganin (10 mg) was dissolved in 1.5 mL of 0.012 M NaIO_4_ aqueous solution and stirred at room temperature for 3 h. Then the reaction solution was cooled to 0 °C, an excess amount of NaBH_4_ (5 mg) was added and kept in this condition for 1 h. The reaction solution was partitioned between water and butanol. The butanol extract was separated by reverse phase HPLC (Luna C18 3 µm, MeOH/H_2_O 8:2 0.1% TFA, 0.5 mL/min) yielding compounds **2** (3.8 mg, t_R_ 2.3 min) and **3** (5.5 mg, t_R_ 20.3 min) in the pure state.

Compound **2**: yellow oil; HRESIMS: *m*/*z* 274.1670 [M − H]^−^ (calcd. for C_13_H_24_NO_5_ *m*/*z* 274.1649). The NMR data of compound **2** agree with literature data [10] (Figures S1 and S2, Supplementary Materials).

Compound **3**: white powder; HRESIMS: *m*/*z* 395.2538 [M − H]^−^ (calcd. for C_19_H_40_O_6_P *m*/*z* 395.2549). The NMR data of compound **3** agree with literature data [10] (Figures S3 and S4, Supplementary Materials).

### 4.3. Synthesis of the Series of Phosphoeleganin-Based Simplified Analogues ***4a**–**9b***

Synthesis of (5*S*,8*S*,9*R*)-tetradecan-5,8,9-triol (**4a**) and (5*S*,8*R*,9*S*)-tetradecan-5,8,9-triol (**4b**): compounds **4a** (80 mg) and **4b** (75 mg) were obtained by carrying out the synthetic protocol reported in literature [11].

Compound **4a**: white powder; HRESIMS: *m*/*z* 269.2101 [M + Na]^+^ (calcd. for C_14_H_30_O_3_Na *m*/*z* 269.2087). The NMR data of compound **4a** agree with literature data [11] (Figures S5 and S6, Supplementary Materials).

Compound **4b**: white powder; HRESIMS: *m*/*z* 269.2103 [M + Na]^+^ (calcd. for C_14_H_30_O_3_Na *m*/*z* 269.2087). The NMR data of compound **4a** agree with literature data [11] (Figures S7 and S8, Supplementary Materials).

Synthesis of compound **5a**: 70 mg (0.284 mmol) of the triol **4a** were dissolved in 17.0 mL of 2,2-dimethoxypropane and a catalytic amount of p-toluenesulfonic acid was added in one portion. The reaction mixture was stirred overnight at rt. Then, a saturated NaHCO_3_ solution quenched the reaction and the desired product **5a** (74.8 mg, 92%) was extracted with EtOAc (2 × 80 mL).

Compound **5a**: colourless oil; ^1^H NMR (CD_3_OD): 0.93 (3H, t, *J* = 7.6 Hz); 1.32 (2H, H-2); 1.32–1.43 (2H, overlapped, H-3); 1.42–1.46 (2H, overlapped, H-4); 3.53 (1H, m, H-5); 1.34 (1H, m, H-6a); 1.68 (1H, m, H-6b); 1.34 (1H, m, H-7a); 1.61 (1H, H-7b); 4.02 (1H, m, H-8); 4.05 (1H, m, H-9); 1.47–1.48 (2H, overlapped, H-10); 1.34 (1H, overlapped, H-11a); 1.54 (1H, m, H-11b); 1.34 (2H, overlapped, H-12); 1.34 (2H, overlapped, H-13); 0.91 (3H, t, *J* = 7.6 Hz, H-14); 1.30 (3H, s, CH_3_, H-1′); 1.39 (3H, s, CH_3_, H-3′); ^13^C NMR (CD_3_OD): 14.1 (CH_3_, C-1); 23.5 (CH_2_, C-2); 28.7 (CH_2_, C-3); 37.9 (CH_2_, C-4); 73.9 (CH, C-5); 34.9 (CH_2_, C-6); 27.5 (CH_2_, C-7); 79.0 (CH, C-8); 79.5 (CH, C-9); 30.4 (CH_2_, C-10); 26.4 (CH_2_, C-11); 32.8 (CH_2_, C-12); 23.7 (CH_2_, C-13); 14.1 (CH_2_, C-14); 108.5 (C, C-2′); 28.5 (CH_3_, C-1′); 25.7 (CH_3_, C-3′); HRESIMS: *m*/*z* 287.2597 [M + H]^+^ (calcd. for C_17_H_35_O_3_ *m*/*z* 287.2581) (Figures S9 and S10, Supplementary Materials).

Synthesis of compound **5b**: the same procedure used for **5a** was adopted to obtain **5b** (64.0 mg, 92%) starting from 60.0 mg (0.243 mmol) of **4b**.

Compound **5b**: colourless oil; ^1^H NMR (CD_3_OD): 0.92 (3H, t, *J* = 7.6 Hz); 1.32 (2H, H-2); 1.32–1.43 (2H, overlapped, H-3); 1.42–1.46 (2H, overlapped, H-4); 3.53 (1H, m, H-5); 1.34 (1H, m, H-6a); 1.68 (1H, m, H-6b); 1.34 (1H, m, H-7a); 1.61 (1H, H-7b); 4.01 (1H, m, H-8); 4.03 (1H, m, H-9); 1.47–1.48 (2H, overlapped, H-10); 1.34 (1H, overlapped, H-11a); 1.54 (1H, m, H-11b); 1.34 (2H, overlapped, H-12); 1.33 (2H, overlapped, H-13); 0.89 (3H, t, *J* = 7.5 Hz, H-14); 1.30 (3H, s, CH_3_, H-1′); 1.39 (3H, s, CH_3_, H-3′); ^13^C NMR (CD_3_OD): 14.1 (CH_3_, C-1); 23.5 (CH_2_, C-2); 28.7 (CH_2_, C-3); 37.6 (CH_2_, C-4); 73.9 (CH, C-5); 34.9 (CH_2_, C-6); 27.6 (CH_2_, C-7); 79.1 (CH, C-8); 79.5 (CH, C-9); 30.5 (CH_2_, C-10); 26.5 (CH_2_, C-11); 32.8 (CH_2_, C-12); 23.6 (CH_2_, C-13); 14.1 (CH_2_, C-14); 109.5 (C, C-2′); 28.5 (CH_3_, C-1′); 25.7 (CH_3_, C-3′); HRESIMS: *m*/*z* 287.2587 [M + H]^+^ (calcd. for C_17_H_35_O_3_ *m*/*z* 287.2581) (Figures S11 and S12, Supplementary Materials).

Synthesis of compound **6a**: acetylation reaction was carried out on 74.8 mg (0.261 mmol) of the acetonide **5a** in pyridine (10.0 mL) as solvent with a great excess of acetic anhydride, leaving the resultant mixture for 18 h at rt. During this time, the reaction was monitored by TLC until the starting material was disappeared. Then, the excess of acetic anhydride was eliminated cooling the reaction mixture at 0 °C in an ice-bath and adding ~25 mL of MeOH. The solvent removal in vacuo afforded compound **6a** that was immediately subjected to hydrolysis reaction.

Compound **6a**: colourless oil; HRESIMS: *m*/*z* 351.2502 [M + Na]^+^ (calcd. for C_19_H_36_O_4_Na *m*/*z* 351.2506); (Figure S13, Supplementary Materials).

Synthesis of compound **6b**: the same procedure used for **6a** was adopted to obtain **6b** starting from 64 mg (0.223 mmol) of **5b**.

Compound **6b**: colourless oil; HRESIMS: *m*/*z* 351.2501 [M + Na]^+^ (calcd. for C_19_H_36_O_4_Na *m*/*z* 351.2506); (Figure S14, Supplementary Materials).

Synthesis of compound **7a**: hydrolysis of the protective acetonide group on the 1,2-diol system was performed dissolving 75 mg of the freshly synthesised **6a** (0.228 mmol) in 18 mL of a mixture MeOH/H_2_O (9:1) adding dropwise 150 μL of HCl 37% (p/p). The reaction mixture was kept at 4°C for 36 h before removing the solvent by rotary evaporator. The raw material was purified by HPLC on C18 reversed phase (Luna 5 μm C18 column, MeOH/H_2_O 7:3 as mobile phase, flow rate 1 mL/min) to yield the acetylated compound **7a** (63.8 mg, 97%).

Compound **7a**: white powder; ^1^H NMR (CD_3_OD): 0.91 (3H, t, *J* = 7.6 Hz, H-1); 1.33 (2H, overlapped, H-2); 1.32 (1H, overlapped, H-3a); 1.28 (1H, m, H-3b); 1.56 (2H, overlapped, H-4); 4.82 (1H, m, H-5); 1.83 (1H, m, H-6a); 1.52 (1H, overlapped, H-6b); 1.68 (1H, m, H-7a); 1.31 (1H, overlapped, H-7b); 3.29 (1H, m, H-8); 3.33 (1H, m, H-9); 1.62 (1H, overlapped, H-10a); 1.35 (1H, overlapped, H-10b); 1.54 (1H, m, H-11a); 1.34 (1H, overlapped, H-11b); 1.33 (2H, overlapped, H-12); 1.34 (2H, overlapped, H-13); 0.91 (3H, t, *J* = 7.6 Hz, H-14); ^13^C NMR (CD_3_OD): 14.1 (CH_3_, C-1); 23.5 (CH_2_, C-2); 28.4 (CH_2_, C-3); 34.7 (CH_2_, C-4); 76.0 (CH, C-5); 31.5 (CH_2_, C-6); 29.4 (CH_2_, C-7); 79.5 (CH, C-8); 79.6 (CH, C-9); 33.5 (CH_2_, C-10); 26.5 (CH_2_, C-11); 32.7 (CH_2_, C-12); 23.5 (CH_2_, C-13); 14.1 (CH_3_, C-14); 173.7 (COCH_3_); 20.9 (COCH_3_); HRESIMS: *m*/*z* 311.2194 [M + Na]^+^ (calcd. for C_16_H_32_O_4_Na *m*/*z* 311.2193) (Figures S15–S17, Supplementary Materials).

Synthesis of compound **7b**: the same procedure used for **7a** was adopted to obtain **7b** (55.4 mg, 97%) starting from 65 mg (0.198 mmol) of **6b**.

Compound **7b**: white powder; ^1^H NMR (CD_3_OD): 0.91 (3H, t, *J* = 7.0 Hz, H-1); 1.33 (2H, overlapped, H-2); 1.34 (2H, overlapped, H-3); 1.58 (2H, overlapped, H-4); 4.82 (2H, m, H-5); 1.60 (2H, overlapped, H-6); 1.39 (1H, overlapped, H-7a); 1.61 (1H, overlapped, H-7b); 3.35 (1H, m, H-8); 3.36 (1H, m, H-9); 1.36 (1H, overlapped, H-10a); 1.56 (1H, overlapped, H-10b); 1.34 (1H, overlapped, H-11a); 1.54 (1H, overlapped, H-11b); 1.34 (2H, overlapped, H-12); 1.35 (2H, overlapped, H-13); 0.91 (3H, t, *J* = 7.0 Hz, H-14); 2.06 (3H, s, COCH_3_) ^13^C NMR (CD_3_OD): 14.4 (CH_3_, C-1); 23.7 (CH_2_, C-2); 32.8 (CH_2_, C-3); 34.8 (CH_2_, C-4); 75.9.0 (CH, C-5); 33.8 (CH_2_, C-6); 28.9 (CH_2_, C-7); 75.3 (CH, C-8); 75.4 (CH, C-9); 33.5 (CH_2_, C-10); 26.7 (CH_2_, C-11); 32.8 (CH_2_, C-12); 23.6 (CH_2_, C-13); 14.3 (CH_3_, C-14); 172.9 (COCH_3_); 21.1 (COCH_3_); HRESIMS: *m*/*z* 311.2191 [M + Na]^+^ (calcd. for C_16_H_32_O_4_Na *m*/*z* 311.2193) (Figures S18–S20, Supplementary Materials).

Synthesis of compounds **8a** and **9a**: 55 mg (0.190 mmol) of compound **7a** in 11.0 mL of acetonitrile were treated with 140 μL (1.400 mmol) of trichloroacetonitrile, following by dropwise addition of 173.0 mg (0.510 mmol) of tetrabutylammonium dihydrogenphosphate, previously solubilised in 4.0 mL of acetonitrile. The reaction was kept under magnetic stirring at rt for 2 h and the solvent removal in vacuo afforded a raw material which was first purified by HPLC on RP-18 (Synergy 4 μm Max-RP column, MeOH:H_2_O 75:25 + 0.1% TFA as mobile phase, flow rate 1 mL/min) and a collected fraction was found to contain a mixture of two isomeric phosphorylated compounds **8a** and **9a** (21.7 mg, 31%). This fraction was further purified by HPLC on RP-18 (Synergy 4 μm Fusion column and MeOH/H_2_O 6:4 + 0.1% TFA as mobile phase, flow rate 1 mL/min) affording compound **8a** (13.6 mg, t_R_ = 27.4 min) and **9a** (8.1 mg, t_R_ = 28.8 min) in pure form.

Compound **8a**: colourless oil; ^1^H NMR (CD_3_OD): 0.91 (3H, t, *J* =6.9 Hz, H-1); 1.31 (2H, overlapped, H-2); 1.28 (1H, overlapped, H-3a); 1.37 (1H, overlapped, H-3b); 1.55 (2H, overlapped, H-4); 4.87 (1H, m, H-5); 1.60 (1H, overlapped, H-6a); 1.82 (1H, overlapped, H-6b); 1.38 (1H, overlapped, H-7a); 1.61 (1H, overlapped, H-7b); 3.67 (1H, ddd, H-8); 4.11 (1H, overlapped, H-9); 1.60 (1H, overlapped H-10a); 1.68 (1H, overlapped, H-10b); 1.41 (1H, overlapped, H-11a); 1.48 (1H, overlapped, H-11b); 1.33 (2H, overlapped, H-12); 1.36 (2H, overlapped, H-13); 0.91 (3H, t, *J* = 6.9 Hz, H-14); 2.02 (3H, s, COCH_3_); ^13^C NMR (CD_3_OD): 14.1 (CH_3_, C-1); 23.4 (CH_2_, C-2); 28.6 (CH_2_, C-3); 34.8 (CH_2_, C-4); 76.0 (CH, C-5); 31.6(CH_2_, C-6); 29.3(CH_2_, C-7); 74.0 (CH,C-8); 82.9 (CH, C-9); 31.5 (CH_2_, C-10); 25.6 (CH_2_, C-11); 32.8 (CH_2_, C-12); 23.4 (CH_2_, C-13); 14.1 (CH_3_, C-14); 173.6 (COCH_3_); 20.9 (COCH_3_). HRESIMS: *m*/*z* 367.1882 [M-H]^-^ (calcd. for C_16_H_32_O_7_P *m*/*z* 367.1880); (Figures S21–S23, Supplementary Materials).

Compound **9a**: colourless oil; ^1^H NMR (CD_3_OD): 0.91 (3H, t, *J* =6.9 Hz, H-1); 1.34 (2H, overlapped, H-2); 1.28 (1H, overlapped, H-3a); 1.32 (1H, overlapped, H-3b); 1.57 (2H, overlapped, H-4); 4.88 (1H, m, H-5); 1.61 (1H, overlapped, H-6a); 1.86 (1H, overlapped, H-6b); 1.65 (1H, overlapped, H-7a); 1.69 (1H, overlapped, H-7b); 4.15 (1H, ddd, H-8); 3.63 (1H, overlapped, H-9); 1.40 (1H, overlapped H-10a); 1.56 (1H, overlapped, H-10b); 1.33 (1H, overlapped, H-11a); 1.56 (1H, overlapped, H-11b); 1.33 (2H, overlapped, H-12); 1.34 (2H, overlapped, H-13); 0.91 (3H, t, *J* = 6.9 Hz, H-14); 2.02 (3H, s, COCH_3_); ^13^C NMR (CD_3_OD): 14.1 (CH_3_, C-1); 23.4 (CH_2_, C-2); 28.4 (CH_2_, C-3); 34.6 (CH_2_, C-4); 76.0 (CH, C-5); 30.7 (CH_2_, C-6); 27.3 (CH_2_, C-7); 82.4 (CH, C-8); 73.8 (CH, C-9); 33.5 (CH_2_, C-10); 26.4 (CH_2_, C-11); 32.7 (CH_2_, C-12); 23.3 (CH_2_, C-13); 14.1 (CH_3_, C-14); 173.7 (COCH_3_); 20.9 (COCH_3_). HRESIMS: *m*/*z* 367.1871 [M − H]^−^ (calcd. for C_16_H_32_O_7_P *m*/*z* 367.1880); (Figures S24–S27, Supplementary Materials).

Synthesis of compounds **8b** and **9b**: the same procedure used for **8a** and **9a** was adopted to obtain **8b** (11.1 mg, t_R_ = 26.9 min) and **9b** (6.5 mg, t_R_ = 27.6 min) (17.8 mg, 31%) starting from 45 mg (0.156 mmol) of compound **7b**.

Compound **8b**: colourless oil; ^1^H NMR (CD_3_OD): 0.91 (3H, t, *J* = 7.0 Hz, H-1); 1.30 (2H, overlapped, H-2); 1.28 (1H, overlapped, H-3a); 1.30 (1H, overlapped, H-3b); 1.56 (2H, overlapped, H-4); 4.90 (1H, m, H-5); 1.65 (1H, overlapped, H-6a); 1.75 (1H, m, H-6b); 1.64 (1H, overlapped, H-7a); 1.66 (1H, overlapped, H-7b); 4.16 (1H, m, H-8); 3.66 (1H, m, H-9); 1.44 (1H, overlapped H-10a); 1.56 (1H, overlapped, H-10b); 1.33 (1H, overlapped, H-11a); 1.55 (1H, overlapped, H-11b); 1.33 (2H, overlapped, H-12); 1.34 (2H, overlapped, H-13); 0.91 (3H, t, *J* = 7.1 Hz, H-14); 2.02 (3H, s, COCH_3_); ^13^C NMR (CD_3_OD): 14.0 (CH_3_, C-1); 23.5 (CH_2_, C-2); 28.5 (CH_2_, C-3); 34.8 (CH_2_, C-4); 75.1 (CH, C-5); 31.2 (CH_2_, C-6); 27.1 (CH_2_, C-7); 82.6 (CH,C-8); 73.4 (CH, C-9); 33.4 (CH_2_, C-10); 26.5 (CH_2_, C-11); 32.6 (CH_2_, C-12); 23.4 (CH_2_, C-13); 14.0 (CH_3_, C-14); 173.1 (COCH_3_); 20.8 (COCH_3_). HRESIMS: *m*/*z* 367.1848 [M − H]^−^ (calcd. for C_16_H_32_O_7_P *m*/*z* 367.1880); (Figures S28–S31, Supplementary Materials).

Compound **9b**: colourless oil; ^1^H NMR (CD_3_OD): 0.91 (3H, t, *J* = 7.0, H-1); 1.34 (2H, overlapped, H-2); 1.30 (1H, overlapped, H-3a); 1.33 (1H, overlapped, H-3b); 1.56 (2H, overlapped, H-4); 4.94 (1H, m, H-5); 1.71 (1H, overlapped, H-6a); 1.76 (1H, overlapped, H-6b); 1.71 (1H, overlapped, H-7a); 1.57 (1H, overlapped, H-7b); 3.71 (1H, m, H-8); 4.17 (1H, m, H-9); 1.39 (1H, overlapped H-10a); 1.52 (1H, overlapped, H-10b); 1.33 (1H, overlapped, H-11a); 1.53 (1H, overlapped, H-11b); 1.30 (2H, overlapped, H-12); 1.34 (2H, overlapped, H-13); 0.90 (3H, t, *J* = 7.0, H-14); 2.02 (3H, s, COCH_3_) ; ^13^C NMR (CD_3_OD): 14.0 (CH_3_, C-1); 23.2 (CH_2_, C-2); 28.3 (CH_2_, C-3); 34.8 (CH_2_, C-4); 74.9 (CH, C-5); 30.4 (CH_2_, C-6); 26.4 (CH_2_, C-7); 73.9 (CH,C-8); 81.7 (CH, C-9); 33.4 (CH_2_, C-10); 26.4 (CH_2_, C-11); 32.7 (CH_2_, C-12); 23.3 (CH_2_, C-13); 14.0 (CH_3_, C-14); 172.7 (COCH_3_); 20.8 (COCH_3_). HRESIMS: *m*/*z* 367.1901 [M − H]^−^ (calcd. for C_16_H_32_O_7_P *m*/*z* 367.1880); (Figures S32–S36, Supplementary Materials).

### 4.4. Enzymatic Assay

#### 4.4.1. Enzymatic Assays with PTP1B

We performed enzymatic assays as previously described [26]. For each assay, an aliquot of human recombinant PTP1B (1–302) was added in the assay buffer (0.075 M β,β-dimethylglutarate pH 7.0 containing 1 mM EDTA, 0.1 mM DTT and a *p*-nitrophenylphosphate (p-NPP) (Chemcruz, Santa Cruz Biotechnology (Santa Cruz, CA, USA)). The reactions were performed at 37 °C in a final volume of 1 mL. After adequate time (30 min) of incubation, the reactions were stopped adding 2 mL of 0.1 M KOH in each sample. The released amount of *p*-nitrophenol, product of the reaction, was determined by measuring the absorbance at 400 nm in a 1 cm pathlength cuvette.

#### 4.4.2. AR Enzymatic Assay

The human recombinant AR, purified to electrophoretic homogeneity as described [27], was used as target enzyme. AR activity was determined at 37 °C as previously described [28] by evaluating the decrease in absorbance at 340 nm linked to NADPH oxidation using a Biochrom Libra S60 spectrophotometer. The standard assay mixture contained 0.25 M sodium phosphate buffer pH 6.8, 0.18 mM NADPH, 0.42 M ammonium sulfate, 0.5 mM EDTA and 4.7 mM D,L-glyceraldehyde. One unit of enzyme activity is the amount that catalyses the conversion of 1 µmol of substrate/min in the above assay conditions. For AR inhibition studies, 0.8 mM L-idose was used as substrate in the conditions described above, in the presence of 10 mU of enzyme and of a final concentration of DMSO of 0.7% (*v*/*v*).

### 4.5. Determination of the IC_50_ Values and Reversibility Assay with PTP1B

The IC_50_ value of compounds against PTP1B was determined by measuring the hydrolysis rate of the enzyme in the presence of a fixed substrate concentration (2.5 mM p-NPP) and increasing inhibitor concentrations (generally 14–16 different concentrations). Each test was carried out in triplicate. Experimental data were normalized respect to control samples and then fitted using a non-linear fitting software, using the following equation:

$$\frac{V_{i}}{V_{0}}=\frac{Max-Min}{1+{\left(\frac{x}{IC_{50}}\right)}^{slope}}+Min$$

where the *Vi/V*_0_ value represents the relative activity calculated in presence of each inhibitor concentration; the maximum and minimum value of the activity are represented by “*Max*” and “*Min*”, respectively; “*x*” is the concentration of the inhibitor; *IC*_50_ is the inhibitor concentration able to decrease the enzymatic activity up to 50%; “*slope*” represents the slope of the curve in the transition zone.

A dilution test was carried out to evaluate if the compound behaves as reversible or irreversible inhibitor of PTP1B. Briefly, an aliquot of the enzyme was diluted with different concentration of inhibitor and the samples incubated at 37 °C for 1 h. After this time, an aliquot of these samples was diluted 500 times in the assay buffer containing 2.5 mM p-NPP, to evaluate whether the enzyme is able to recover its activity since the final concentration of inhibitor is now far away from its IC_50_ value. Control test was carried out diluting the enzyme with an equal volume of deionised water. All tests were performed in triplicate. Obtained data were normalized respect to the control experiment and reported in the figures as mean value +/− S.D.

### 4.6. Determination of the Mechanism of Inhibition on PTP1B

The inhibitory mechanism of tested compounds was determined studying the dependence between the main kinetics parameters (K_M_ and V_max_) and the inhibitors concentrations. Briefly, K_M_ and V_max_ values were calculated measuring the hydrolysis rate of PTP1B in the absence/presence of different inhibitor concentrations, while increasing substrate content. Data obtained were fitted using Michaelis-Menten equation and a non-linear fitting software. Then, data were analysed using the double reciprocal plot (Lineweaver-Burk method).

### 4.7. Cell Cultures

Murine myoblasts (C2C12) and human liver cells were purchased from the American Type Culture Collection (ATCC, Manassas, VA, USA). C2C12 cells were routinely cultured in Dulbecco’s Modified Eagle’s Medium (DMEM)-high glucose (4500 mg/L) supplemented with 10% Foetal Bovine Serum (FBS, Euroclone, Milan, Italy), 2 mM glutamine, 100 U/mL penicillin, and 100 μg/mL streptomycin (Sigma-Aldrich, St. Louis, MO, USA). Cells were incubated at 37 °C in humidified atmosphere with 5% CO_2_. Myoblast differentiation was induced by adding differentiation medium (DMEM containing 2% horse serum) to 80% confluent myoblasts and incubating them for five days.

### 4.8. Insulin Signalling Pathway Analysis

C2C12 cells were plated on P35 dishes, grown until 80% confluence and then incubated in the presence of differentiation medium (DMEM-high glucose (4500 mg/L) supplemented with 2% Horse serum (HS, Euroclone), 2 mM glutamine, 100 U/mL penicillin, and 100 μg/mL streptomycin) for 4 days. After this time, cell plates were washed with PBS and cells stimulated with 10 nM insulin, compound **3** or insulin-compound **3** combination for 30 min. After cell plates were washed with cold PBS solution and lysed using 1X Leammli sample buffer. Samples were store at 4 °C for 15 min and then protein solutions were collected and boiled for 5 min. Proteins were analysed by SDS-PAGE and then transferred on a PVDF membrane by western blot. Phosphorylation status of insulin receptor and the kinase Akt was evaluated using specific antibodies (pIR β subunit, Y1162/1163 (sc-25103-R) and β-actin, clone C-4 (sc-47778) were from Santa Cruz Biotechnology (Santa Cruz, CA, USA); IR β subunit, clone CT-3 (MABS65) was from Merck-Millipore (Burlington, MA, USA). Akt (9272S) and p-Akt (9271S) antibodies were from Cell Signalling Technology (Danvers, MA, USA). Secondary antibodies were from Santa Cruz Biotechnology (Santa Cruz, CA, USA). Detection was performed using Clarity western ECL substrates (Bio-Rad Laboratories, Inc.).

### 4.9. Glucose Uptake Assay

To evaluate glucose uptake, C2C12 cells were grown until 80% confluence and then incubated in starvation medium for 24 h. After this time, cells were stimulated with 10 nM insulin (Humalog Lispro, Eli Lilly), 20 µM compound **3** or **3**-insulin combination for 30 min and then incubated in the presence of 40 µM of 2-NBDG (Invitrogen) for 3 h. Then, cells were washed with PBS, tripsinized, pelleted by centrifugation (1000× *g* for 5 min), then suspended in 500 µL of PBS. The amount of 2-NBDG uploaded by cells was determined analysing cells by using a flow cytometer apparatus (FACSCanto II, BD Biosciences, San Jose, CA, USA). For each sample 1 × 10^4^ events were acquired. Data obtained were then analysed with FlowJo software. Cells autofluorescence values were determined before sample analysis and subtracted to each sample.

### 4.10. Statistical Analysis

The results were expressed as mean ± SD. The differences between the experimental and control groups were compared using one way ANOVA followed by Tukey’s HSD test for pairwise comparison. All statistical analyses were performed using OriginPro 8.0 2021 software. The *p* value < 0.05 was considered statistically significant. * *p* < 0.05; ** *p* < 0.01.

## 5. Conclusions

Using phosphoeleganin (1) as bioactive molecule model, we have obtained, both by its chemical manipulation and by synthesis, several small fragments inspired to the most functionalized part of the parent bioactive NP structure, in an easy and inexpensive way, even in terms of atom economy. Pharmacological screening of the obtained fragments library highlighted that all compounds in the series significantly lost the activity against AR. However, our studies permit the highlighting of the phosphate moiety and an aliphatic portion of suitable size, as crucial requirements for the AR inhibition. On the other hand, we demonstrated that the chemical scaffold equipped with a monophosphate ester group is a viable and promising chemotype for human PTP1B enzyme inhibition, even considering that it can be produced easily, cheaply, and sustainably through the proposed synthetic scheme. Noteworthy, the two most potent PTP1B inhibitors in the series, compounds **3** and **8a**, exhibit the same regiochemistry and absolute configuration as phosphoeleganin at the 1,2-monophosphorylated diol system; thus, a certain position in the chain and the spatial orientation of the phosphate group appears to be a key requirement for enhancing PTP1B inhibition. This preliminary study outlines the semisynthetic compound **3** and the synthetic analogue **8a** as structures to be decorated and optimized to acquire drugs with a more powerful inhibitory activity on PTP1B and at the same time able to inhibit AR, thus regaining the dual inhibitory activity toward PTP1B and AR showed by phosphoeleganin.

## Figures and Tables

**Table 1:** IC_50_ values determined on PTP1B.

Compounds	IC_50_ (µM)
**Phosphoeleganin**	0.7 ± 0.1
**3**	6.7 ± 3.3
**8a**	16.0 ± 2.0
**9a**	83.0 ± 15.0
**8b**	37.1 ± 6.5
**9b**	63.0 ± 14.0

## Data Availability

Data is contained within article and Supplementary Materials.

## Supplementary Materials

The following are available online at https://www.mdpi.com/article/10.3390/ph15030325/s1, Figure S1. ^1^H NMR spectrum in CD_3_OD (700 MHz) of compound **2**; Figure S2. HR-ESIMS spectrum of compound **2**; Figure S3. ^1^H NMR spectrum in CD_3_OD (700 MHz) of compound **3**; Figure S4. HR-ESIMS spectrum of compound **3**; Figure S5. ^1^H NMR spectrum in CD_3_OD (700 MHz) of compound **4a**; Figure S6. HR-ESIMS spectrum of compound **4a**; Figure S7. ^1^H NMR spectrum in CD_3_OD (700 MHz) of compound **4b**; Figure S8. HR-ESIMS spectrum of compound **4b**; Figure S9. ^1^H NMR spectrum in CD_3_OD (700 MHz) of compound **5a**; Figure S10. HR-ESIMS spectrum of compound **5a**; Figure S11. ^1^H NMR spectrum in CD_3_OD (700 MHz) of compound **5b**; Figure S12. HR-ESIMS spectrum of compound **5b**; Figure S13. HR-ESIMS spectrum of compound **6a**; Figure S14. HR-ESIMS spectrum of compound **6b**; Figure S15. ^1^H NMR spectrum in CD_3_OD (700 MHz) of compound **7a**; Figure S16. ^13^C NMR spectrum in CD_3_OD (175 MHz) of compound **7a**; Figure S17. HR-ESIMS spectrum of compound **7a**; Figure S18. ^1^H NMR spectrum in CD_3_OD (700 MHz) of compound **7b**; Figure S19. ^13^C NMR spectrum in CD_3_OD (175 MHz) of compound **7b**; Figure S20. HR-ESIMS spectrum of compound **7b**; Figure S21. ^1^H NMR spectrum in CD_3_OD (700 MHz) of compound **8a**; Figure S22. ^1^H-^1^H COSY spectrum in CD_3_OD (700 MHz) of compound **8a**; Figure S23. HR-ESIMS spectrum of compound **8a**; Figure S24. ^1^H NMR spectrum in CD_3_OD (700 MHz) of compound **9a**; Figure S25. ^1^H-^1^H COSY spectrum in CD_3_OD (700 MHz) of compound **9a**; Figure S26. ^1^H-^13^C HSQC spectrum in CD_3_OD (700 MHz) of compound **9a**; Figure S27. HR-ESIMS spectrum of compound **9a**; Figure S28. ^1^H NMR spectrum in CD_3_OD (700 MHz) of compound **8b**; Figure S29. ^1^H-^1^H COSY spectrum in CD_3_OD (700 MHz) of compound **8b**; Figure S30. ^1^H-^13^C HSQC spectrum in CD_3_OD (700 MHz) of compound **8b**; Figure S31. HR-ESIMS spectrum of compound **8b**; Figure S32. ^1^H NMR spectrum in CD_3_OD (700 MHz) of compound **9b**; Figure S33. ^1^H-^1^H COSY spectrum in CD_3_OD (700 MHz) of compound **9b**; Figure S34. ^1^H-^1^H TOCSY spectrum in CD_3_OD (700 MHz) of compound **9b**; Figure S35. ^1^H-^13^C HSQC spectrum in CD_3_OD (700 MHz) of compound **9b**; Figure S36. HR-ESIMS spectrum of compound **9b**; Table S1. Effects of compounds **2**, **3**, **4a**, **4b**, and **8a**–**9b** on human AR.
