# Supplementary material for: Identifying Human PTP1B Enzyme Inhibitors from Marine Natural Products: Perspectives for Developing of Novel Insulin-Mimetic Drugs
Source: Pharmaceuticals (Basel). 2022 Mar 8;15(3):325. doi: 10.3390/ph15030325 (PMC8950868; doi:10.3390/ph15030325)
Supplement: Supplementary file 1 [file pharmaceuticals-15-00325-s001.zip › pharmaceuticals-1586145-supplementary.pdf]

Supplementary material

Table of contents

Figure S1. ^{1}H NMR spectrum in CD_3OD (700 MHz) of compound **2** 2
Figure S2.HR-ESIMS spectrum of compound **2** 2
Figure S3.^{1}H NMR spectrum in CD_3OD (700 MHz) of compound **3** 3
Figure S4.HR-ESIMS spectrum of compound **3** 3
Figure S5.^{1}H NMR spectrum in CD_3OD (700 MHz) of compound **4a** 4
Figure S6.HR-ESIMS spectrum of compound **4a** 4
Figure S7.^{1}H NMR spectrum in CD_3OD (700 MHz) of compound **4b** 5
Figure S8.HR-ESIMS spectrum of compound **4b** 5
Figure S9.^{1}H NMR spectrum in CD_3OD (700 MHz) of compound **5a** 6
Figure S10.HR-ESIMS spectrum of compound **5a** 6
Figure S11.^{1}H NMR spectrum in CD_3OD (700 MHz) of compound **5b** 7
Figure S12.HR-ESIMS spectrum of compound **5b** 7
Figure S13.HR-ESIMS spectrum of compound **6a** 8
Figure S14.HR-ESIMS spectrum of compound **6b** 8
Figure S15.^{1}H NMR spectrum in CD_3OD (700 MHz) of compound **7a** 9
Figure S16.^{13}C NMR spectrum in CD_3OD (175 MHz) of compound **7a** 9
Figure S17.HR-ESIMS spectrum of compound **7a** 10
Figure S18.^{1}H NMR spectrum in CD_3OD (700 MHz) of compound **7b** 10
Figure S19.^{13}C NMR spectrum in CD_3OD (175 MHz) of compound **7b** 11
Figure S20.HR-ESIMS spectrum of compound **7b** 11
Figure S21.^{1}H NMR spectrum in CD_3OD (700 MHz) of compound **8a** 12
Figure S22.^{1}H-^{1}H COSY spectrum in CD_3OD (700 MHz) of compound **8a** 12
Figure S23.HR-ESIMS spectrum of compound **8a** 13
Figure S24.^{1}H NMR spectrum in CD_3OD (700 MHz) of compound **9a** 13
Figure S25.^{1}H-^{1}H COSY spectrum in CD_3OD (700 MHz) of compound **9a** 14
Figure S26.^{1}H-^{13}C HSQC spectrum in CD_3OD (700 MHz) of compound **9a** 14
Figure S27.HR-ESIMS spectrum of compound **9a** 15
Figure S28.^{1}H NMR spectrum in CD_3OD (700 MHz) of compound **8b** 15
Figure S29.^{1}H-^{1}H COSY spectrum in CD_3OD (700 MHz) of compound **8b** 16
Figure S30.^{1}H-^{13}C HSQC spectrum in CD_3OD (700 MHz) of compound **8b** 16
Figure S31.HR-ESIMS spectrum of compound **8b** 17
Figure S32.^{1}H NMR spectrum in CD_3OD (700 MHz) of compound **9b** 17
Figure S33.^{1}H-^{1}H COSY spectrum in CD_3OD (700 MHz) of compound **9b** 18
Figure S34.^{1}H-^{1}H TOCSY spectrum in CD_3OD (700 MHz) of compound **9b** 18
Figure S35.^{1}H-^{13}C HSQC spectrum in CD_3OD (700 MHz) of compound **9b** 19
Figure S36.HR-ESIMS spectrum of compound **9b** 19
Table S1. Effects of compounds **2**, **3**, **4a**, **4b**, and **8a-9b** on human AR 20

Figure S1.^{1}H NMR spectrum in CD_3OD (700 MHz) of compound **2**

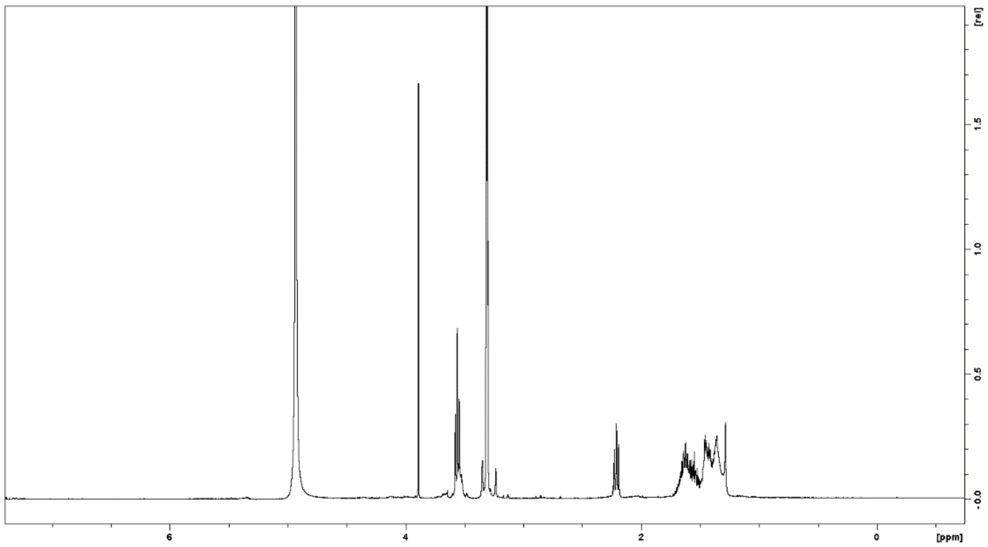

Figure S2.HR-ESIMS spectrum of compound **2**

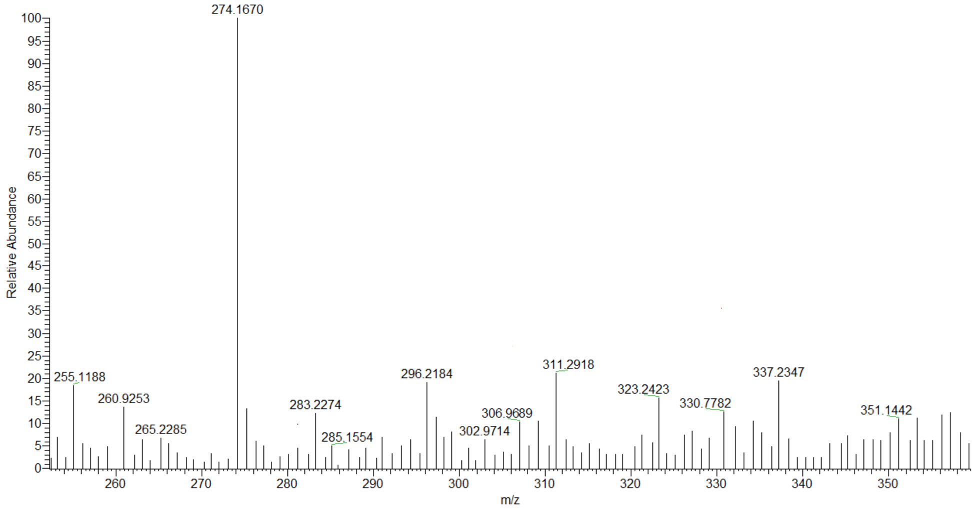

Figure S3.^{1}H NMR spectrum in CD_3OD (700 MHz) of compound **3**

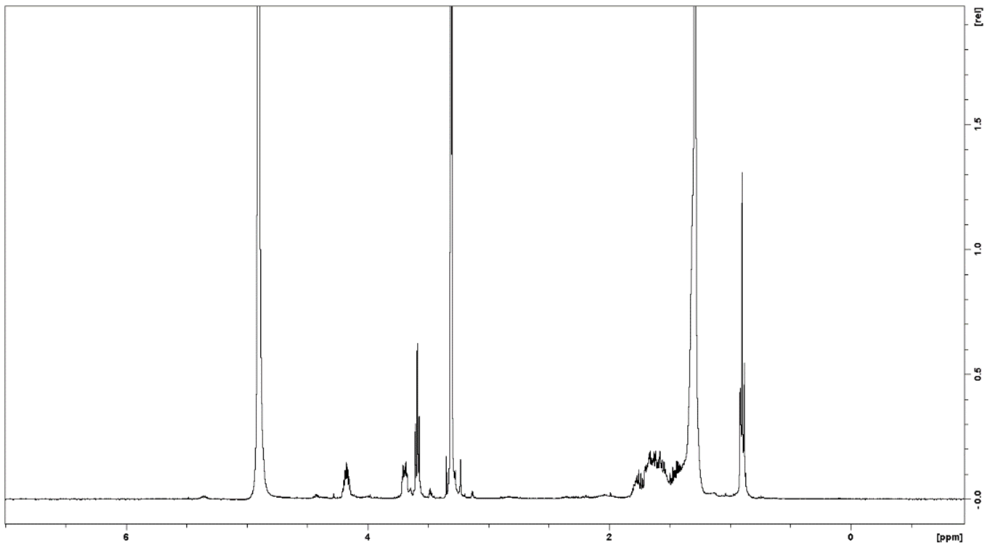

Figure S4.HR-ESIMS spectrum of compound **3**

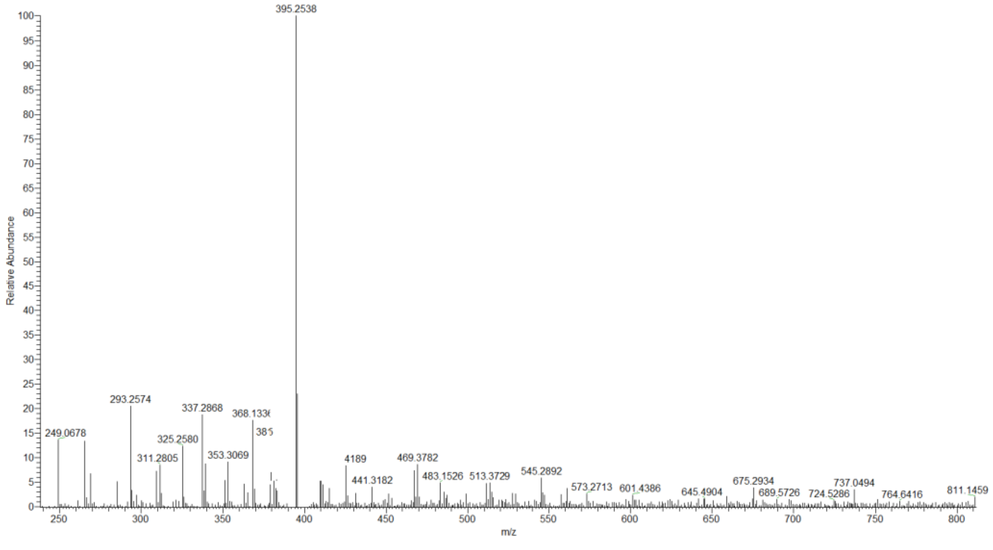

Figure S5.¹H NMR spectrum in CD_3OD (700 MHz) of compound **4a**

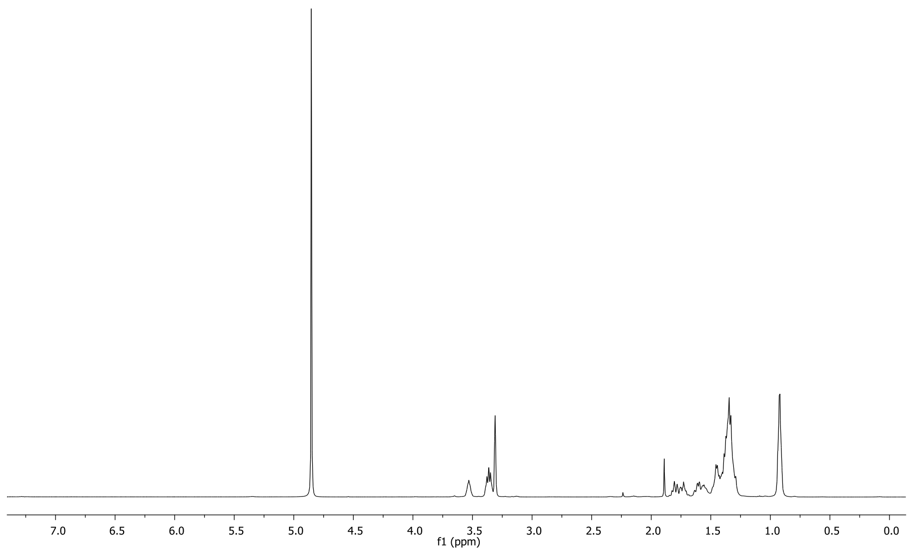

Figure S6.HR-ESIMS spectrum of compound **4a**

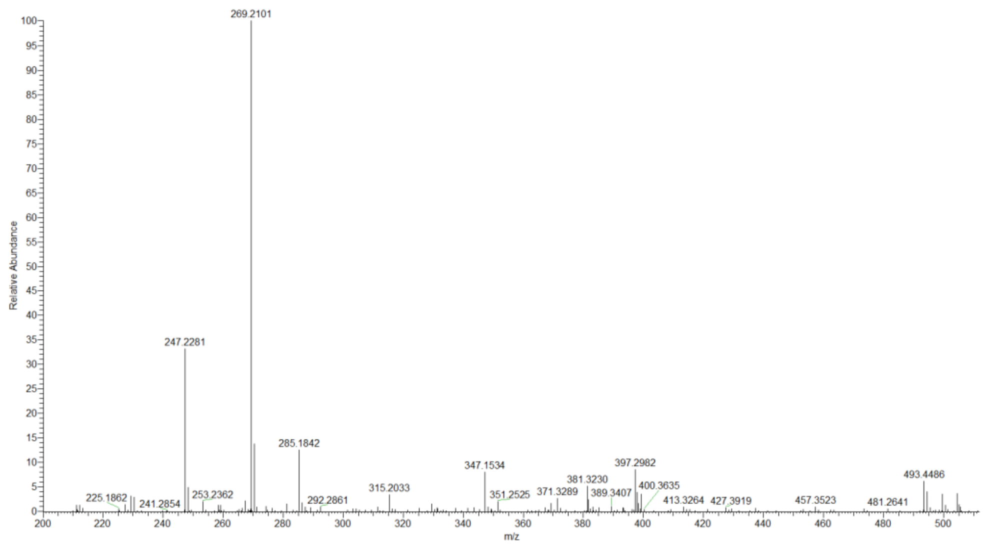

Figure S7.¹H NMR spectrum in CD_3OD (700 MHz) of compound **4b**

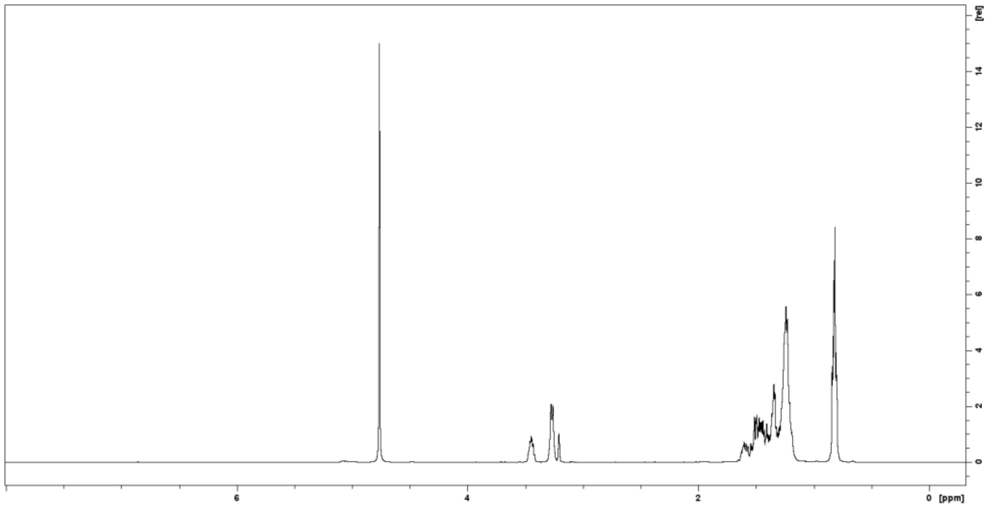

Figure S8.HR-ESIMS spectrum of compound **4b**

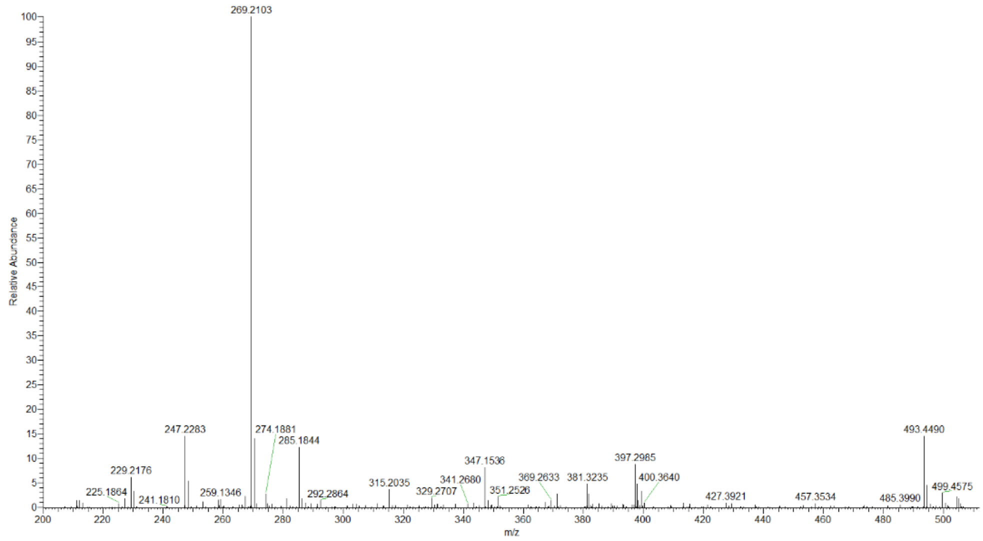

Figure S9.¹H NMR spectrum in CD_3OD (700 MHz) of compound **5a**

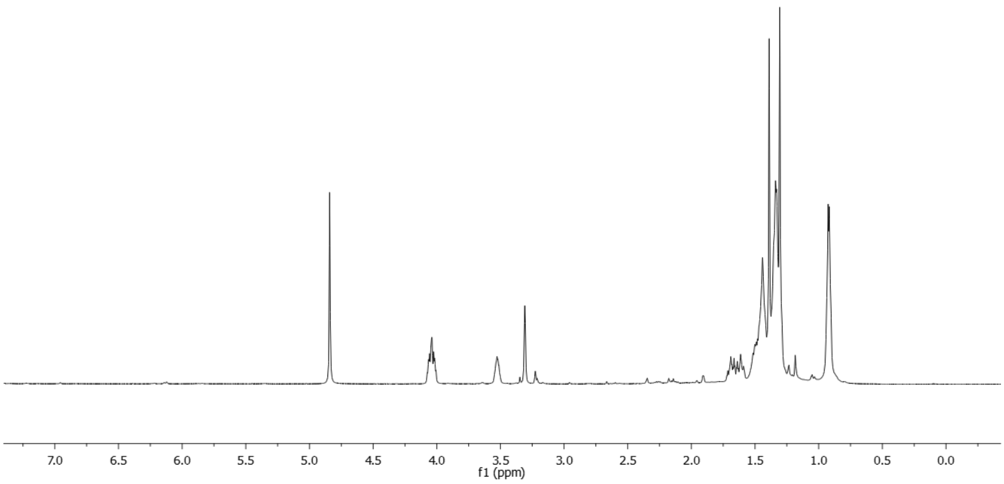

Figure S10.HR-ESIMS spectrum of compound **5a**

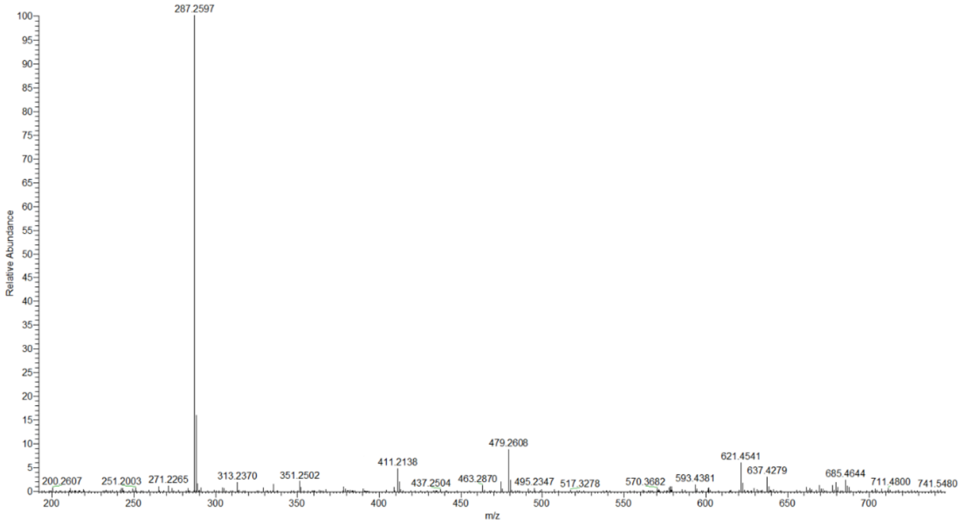

Figure S11.^{1}H NMR spectrum in CD_3OD (700 MHz) of compound **5b**

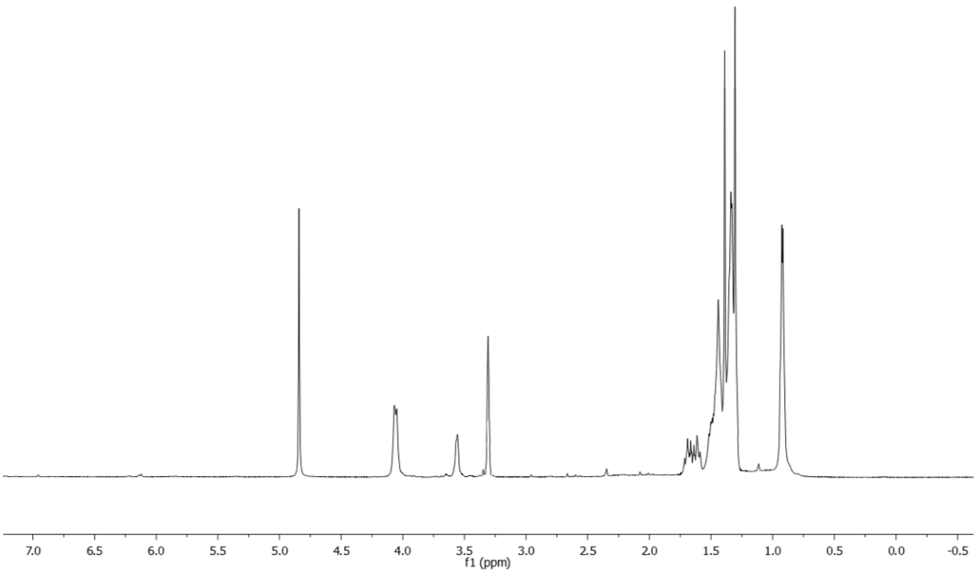

Figure S12.HR-ESIMS spectrum of compound **5b**

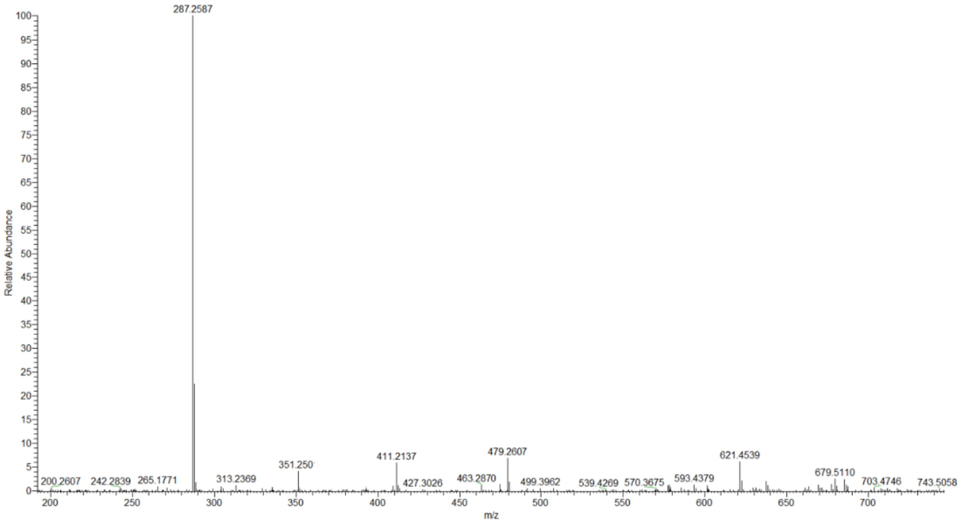

Figure S13.HR-ESIMS spectrum of compound **6a**

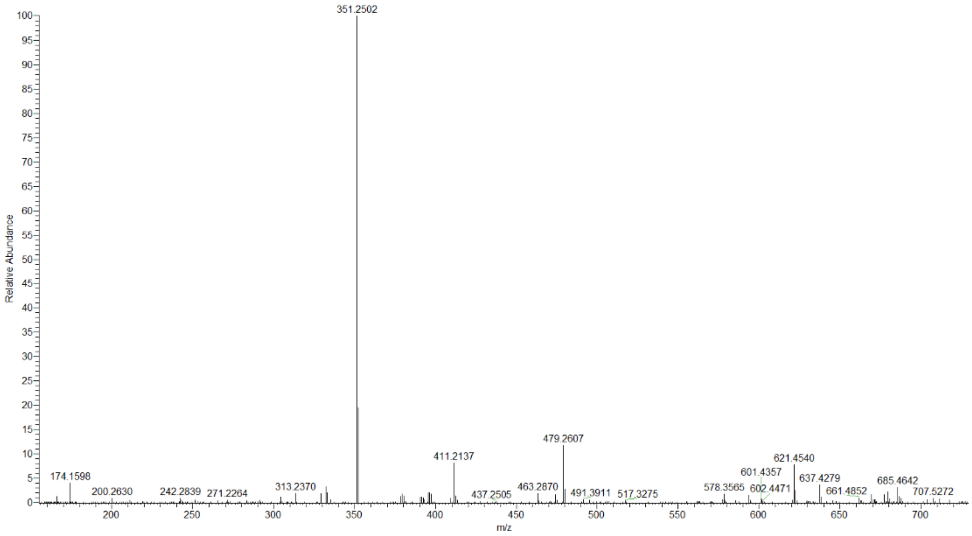

Figure S14.HR-ESIMS spectrum of compound **6b**

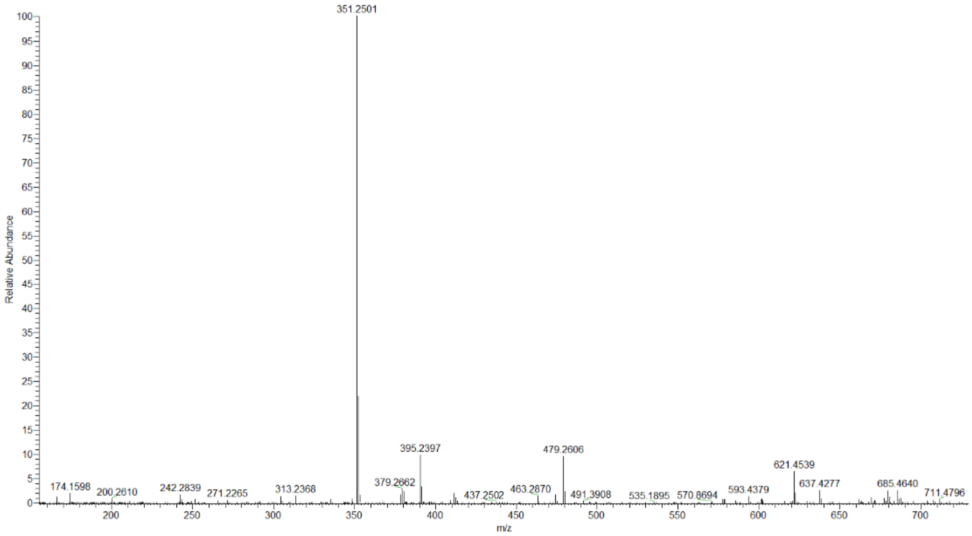

Figure S15.^{1}H NMR spectrum in CD_3OD (700 MHz) of compound **7a**

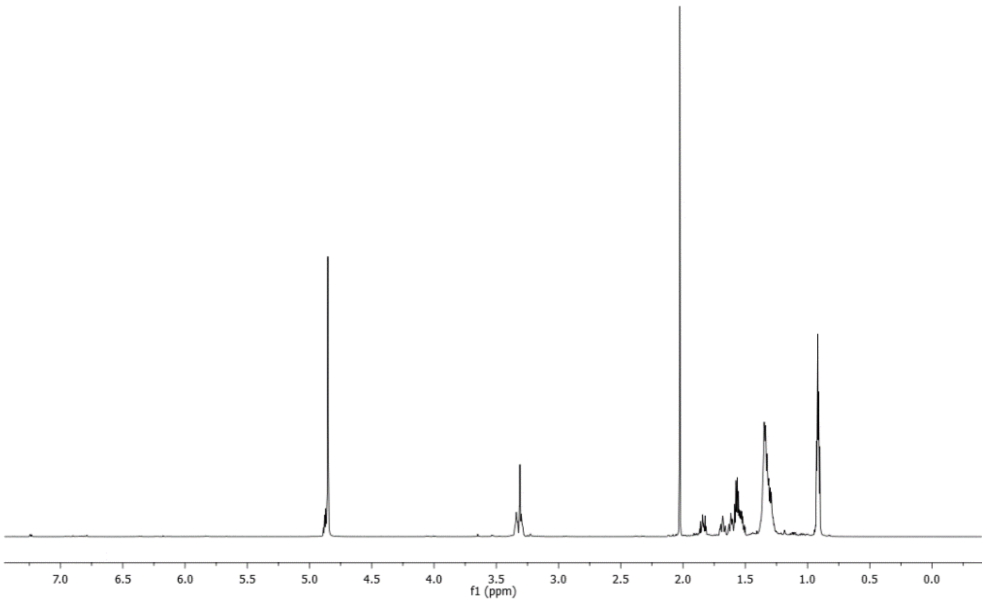

Figure S16.^{13}C NMR spectrum in CD_3OD (175 MHz) of compound **7a**

Figure S17.HR-ESIMS spectrum of compound **7a**

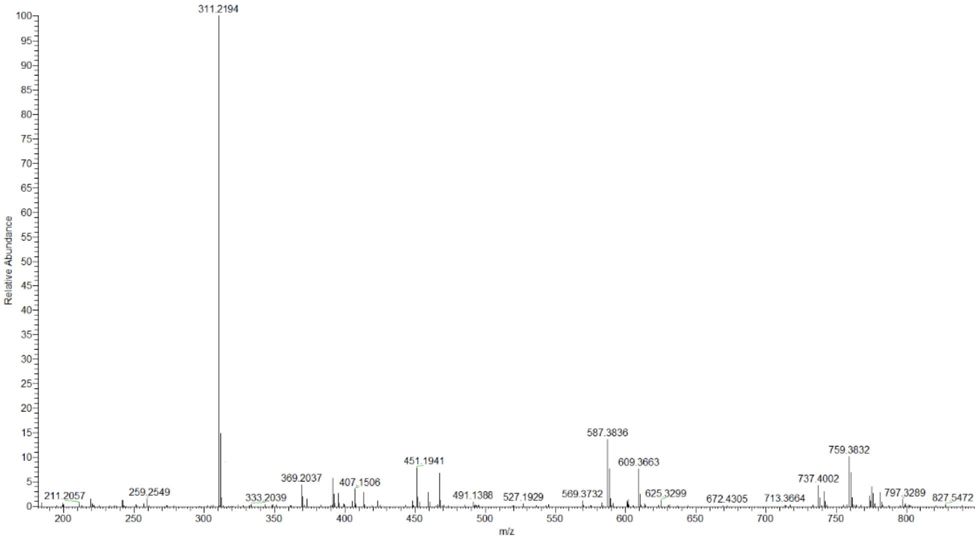

Figure S18.^{1}H NMR spectrum in CD_3OD (700 MHz) of compound **7b**

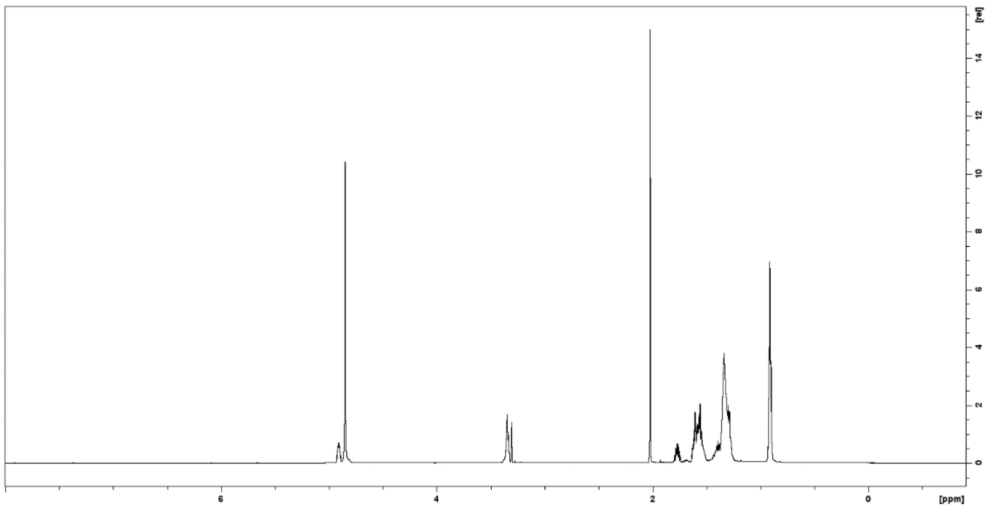

Figure S19.^{13}C NMR spectrum in CD_3OD (175 MHz) of compound **7b**

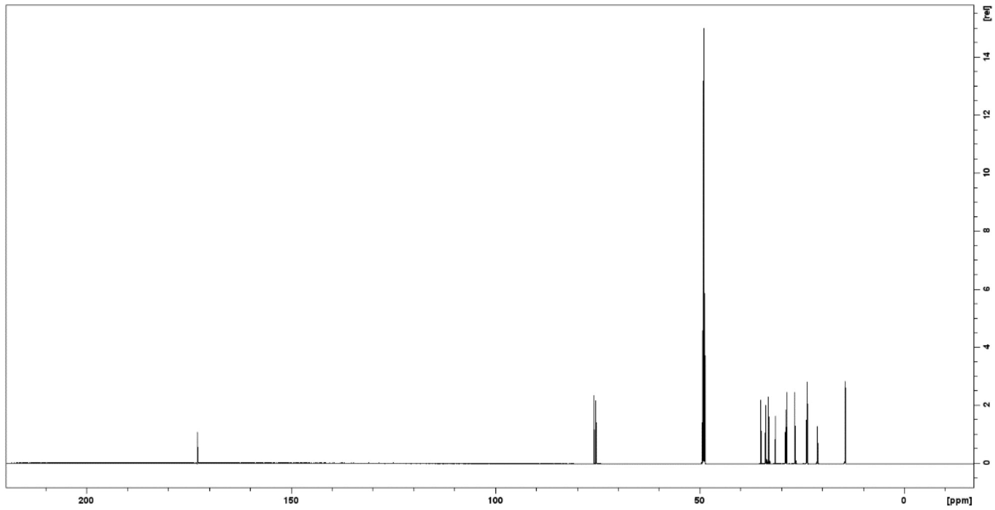

Figure S20.HR-ESIMS spectrum of compound **7b**

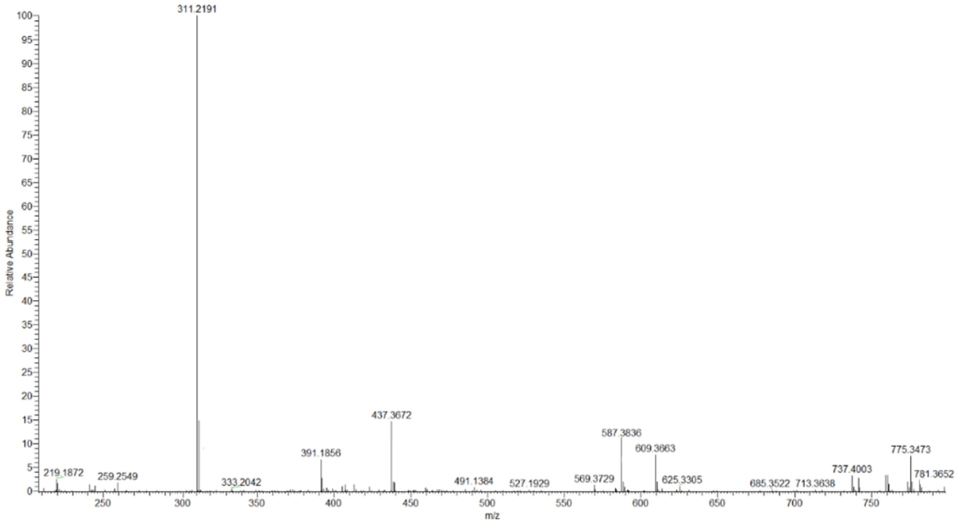

Figure S21.^{1}H NMR spectrum in CD_3OD (700 MHz) of compound **8a**

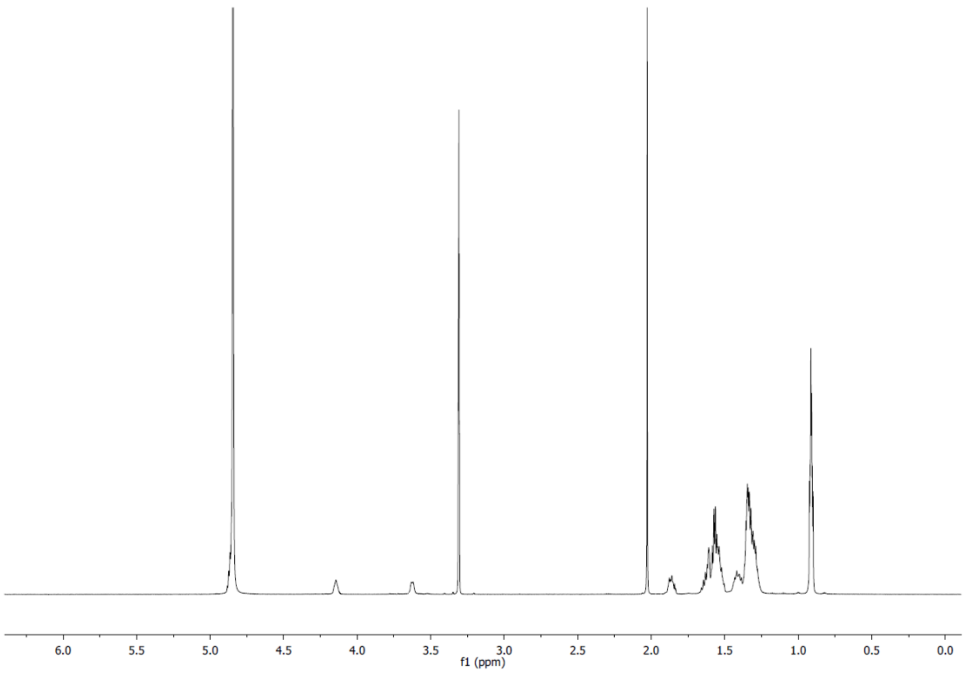

Figure S22.^{1}H-^{1}H COSY spectrum in CD_3OD (700 MHz) of compound **8a**

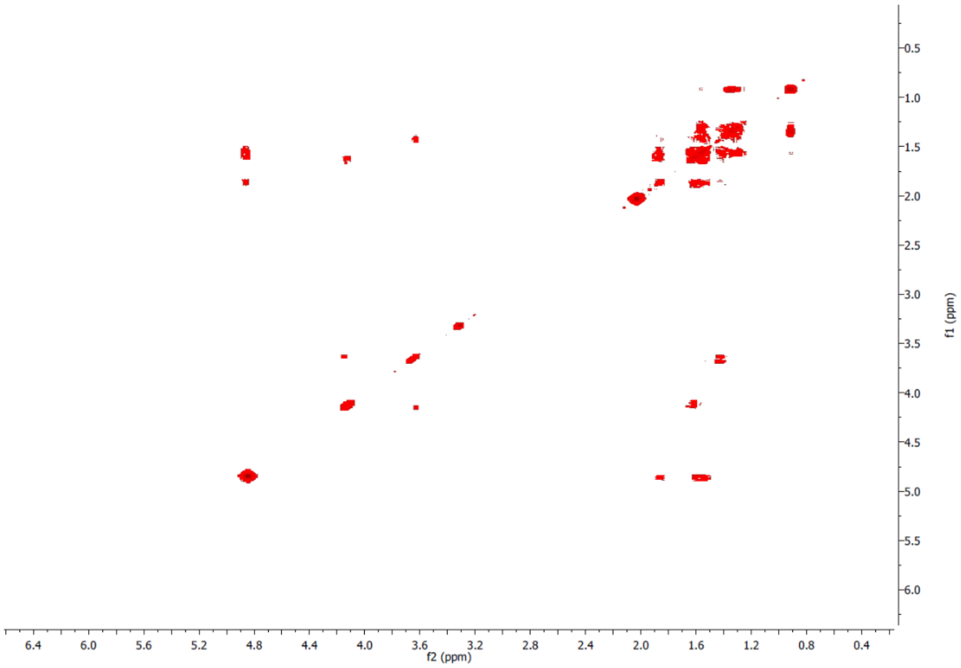

Figure S23.HR-ESIMS spectrum of compound **8a**

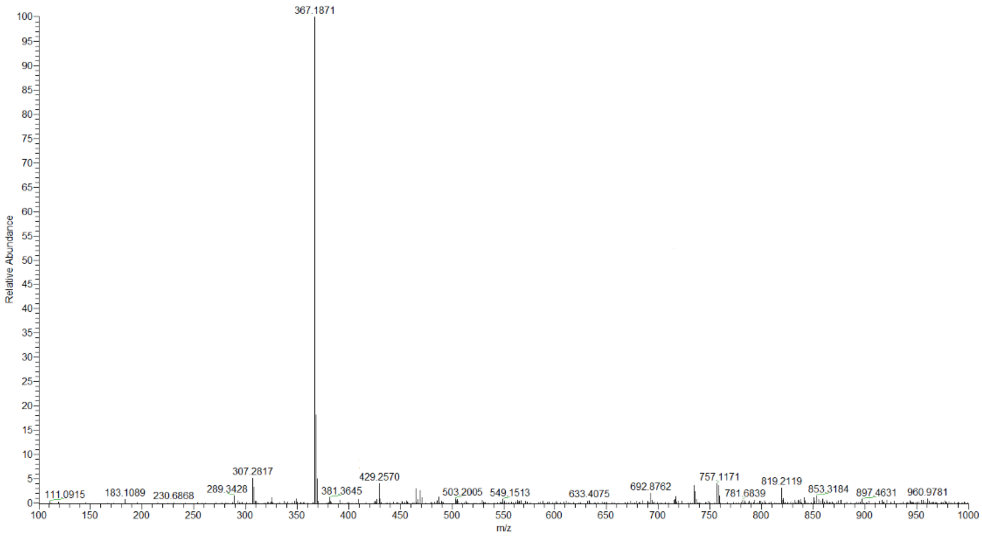

Figure S24.^{1}H NMR spectrum in CD_3OD (700 MHz) of compound **9a**

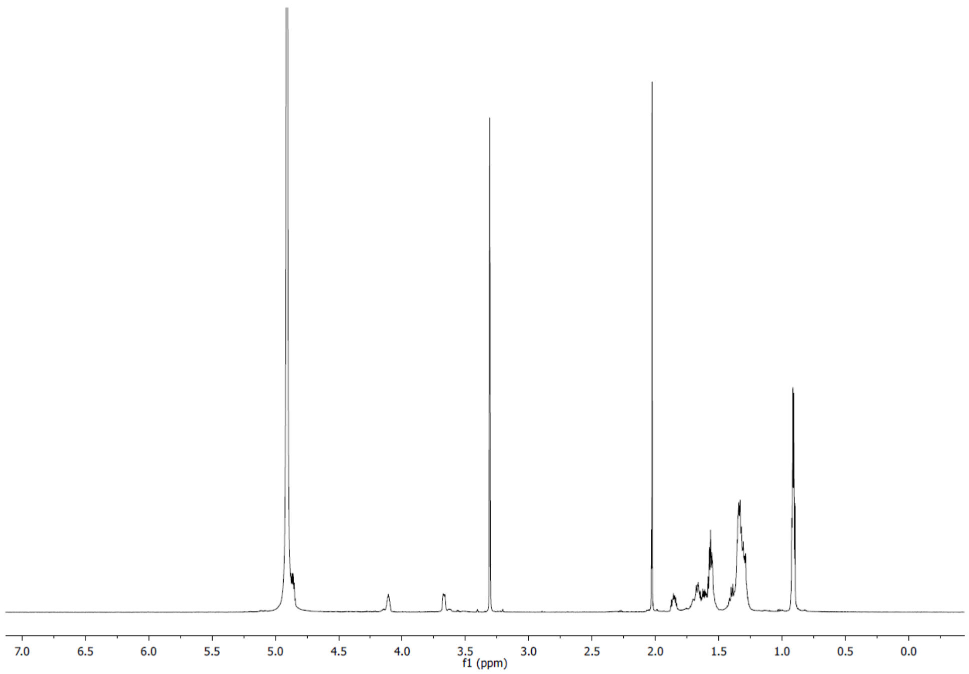

Figure S25.^{1}H-^{1}H COSY spectrum in CD_3OD (700 MHz) of compound **9a**

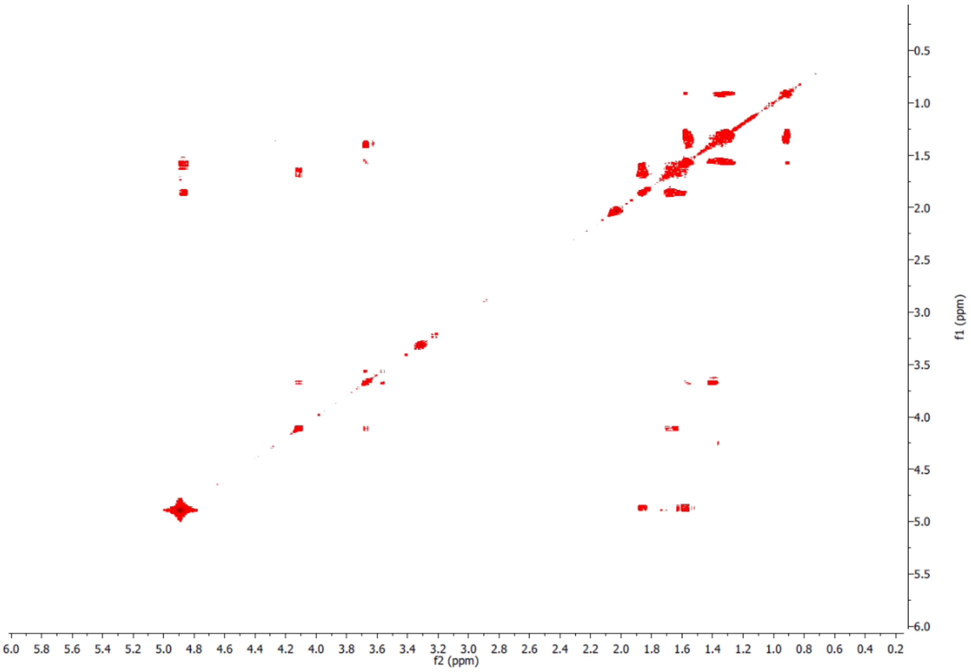

Figure S26.^{1}H-^{13}C HSQC spectrum in CD_3OD (700 MHz) of compound **9a**

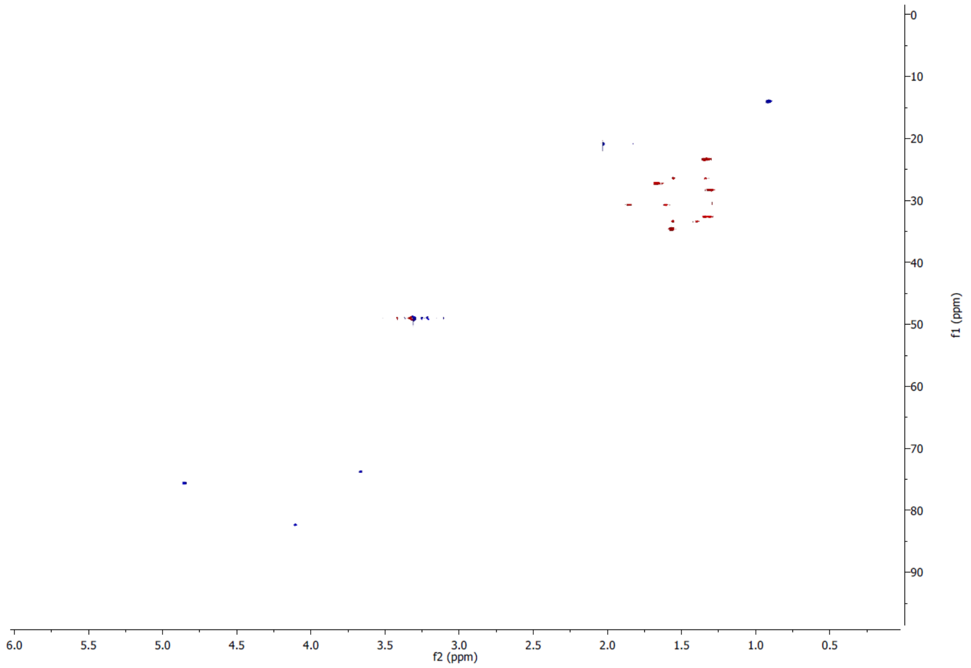

Figure S27.HR-ESIMS spectrum of compound **9a**

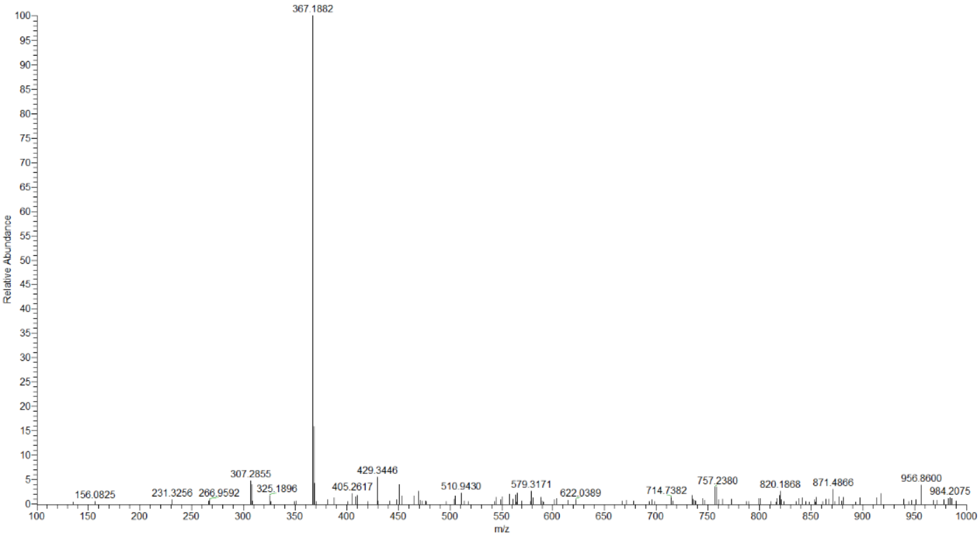

Figure S28.^{1}H NMR spectrum in CD_3OD (700 MHz) of compound **8b**

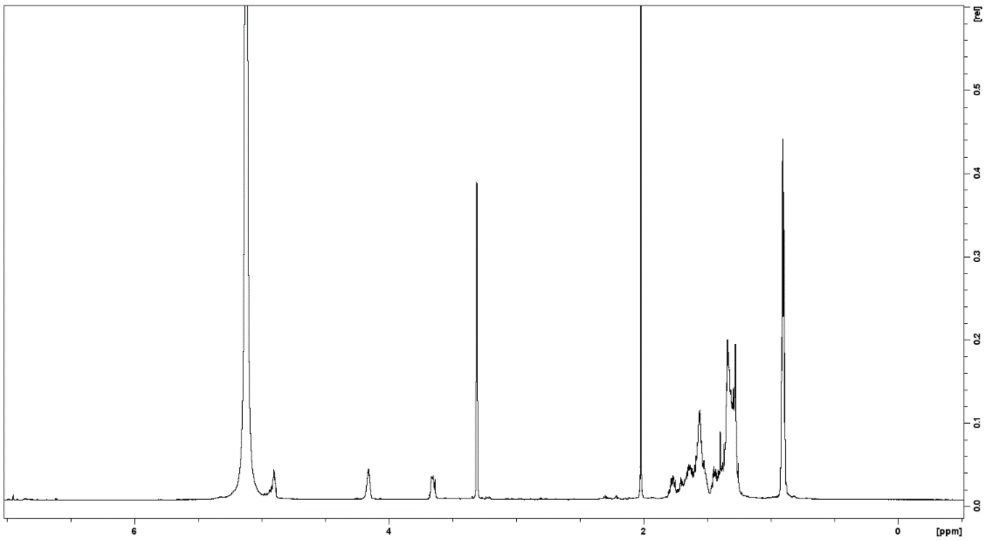

Figure S29. ^{1}H-^{1}H COSY spectrum in CD_3OD (700 MHz) of compound **8b**

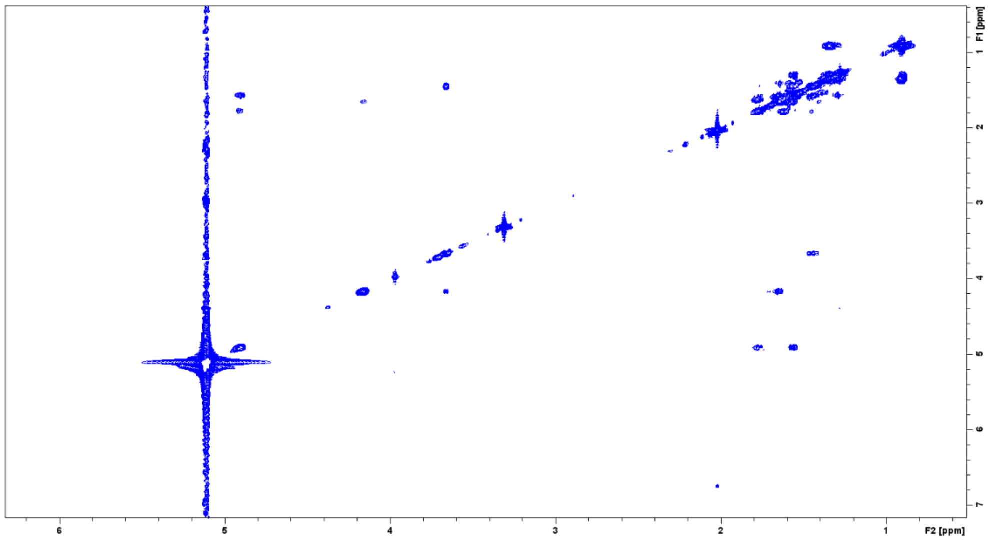

Figure S30. ^{1}H-^{13}C HSQC spectrum in CD_3OD (700 MHz) of compound **8b**

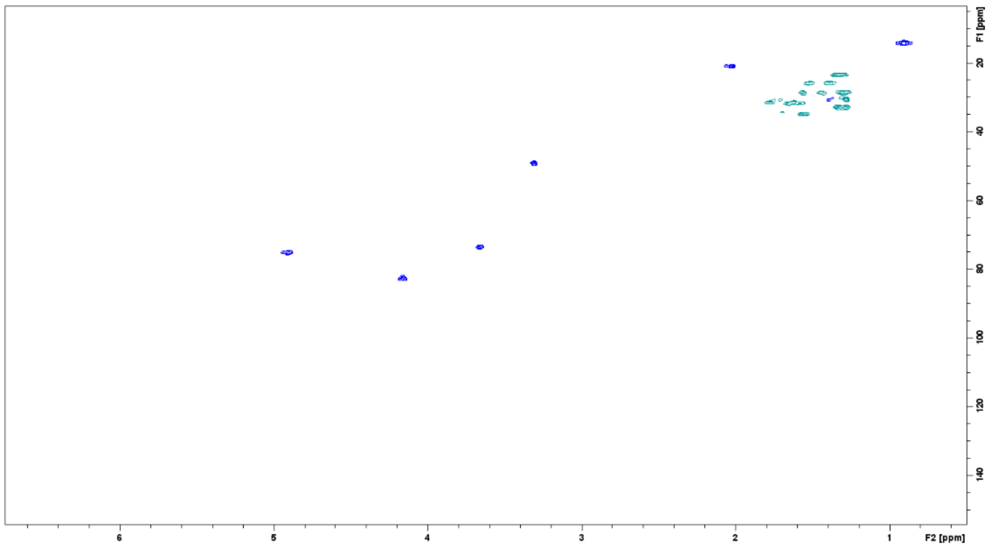

Figure S31. HR-ESIMS spectrum of compound **8b**

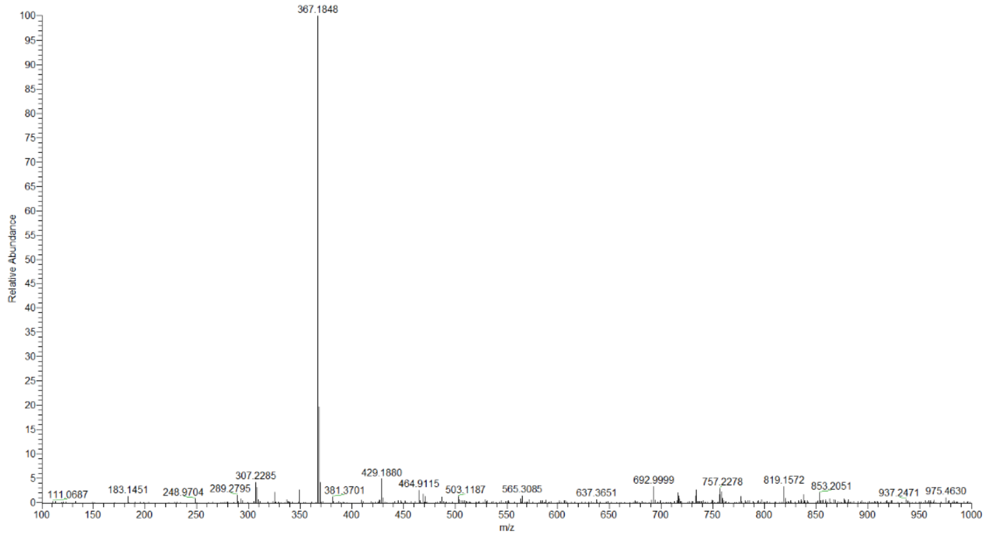

Figure S32. ^{1}H NMR spectrum in CD_3OD (700 MHz) of compound **9b**

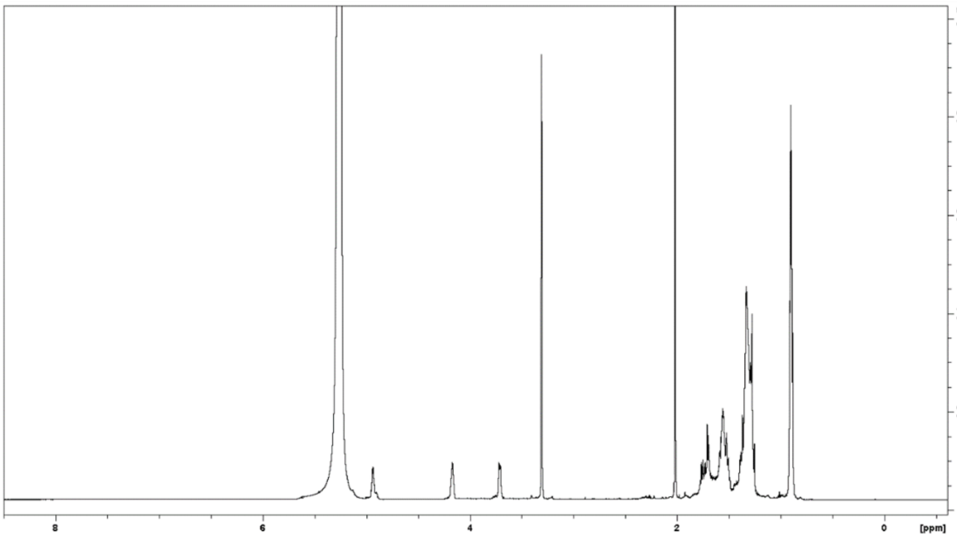

Figure S33. ^{1}H-^{1}H COSY spectrum in CD_3OD (700 MHz) of compound **9b**

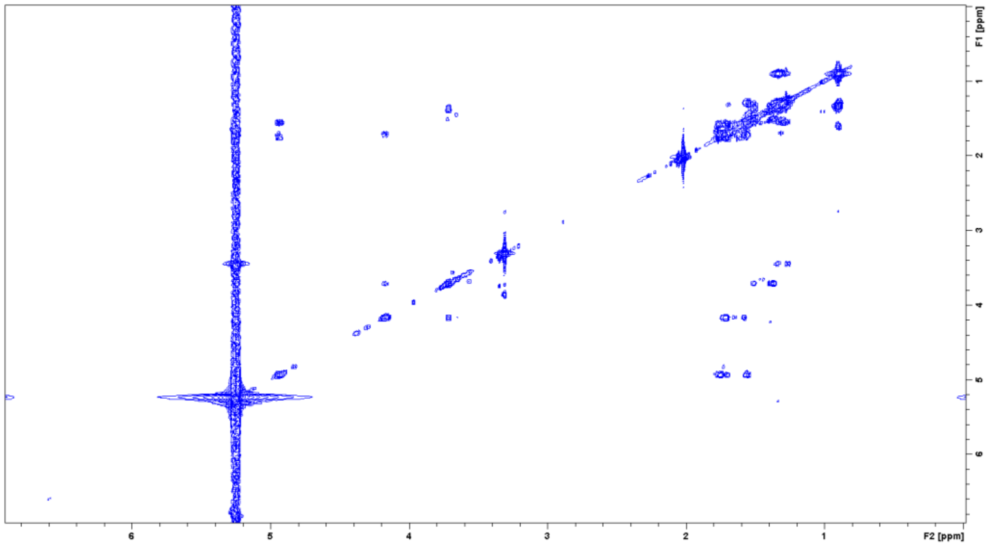

Figure S34. ^{1}H-^{1}H TOCSY spectrum in CD_3OD (700 MHz) of compound **9b**

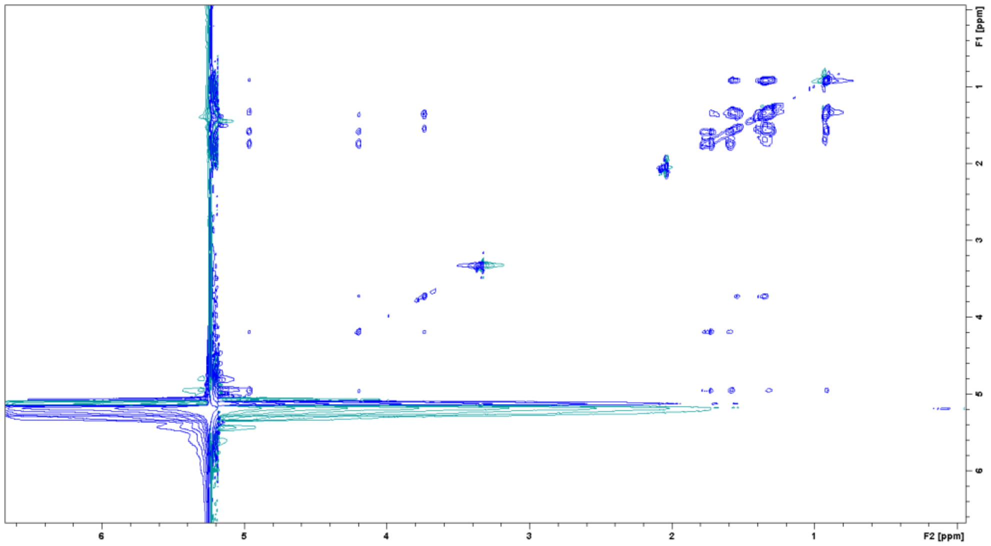

Figure S35.^{1}H-^{13}C HSQC spectrum in CD_3OD (700 MHz) of compound **9b**

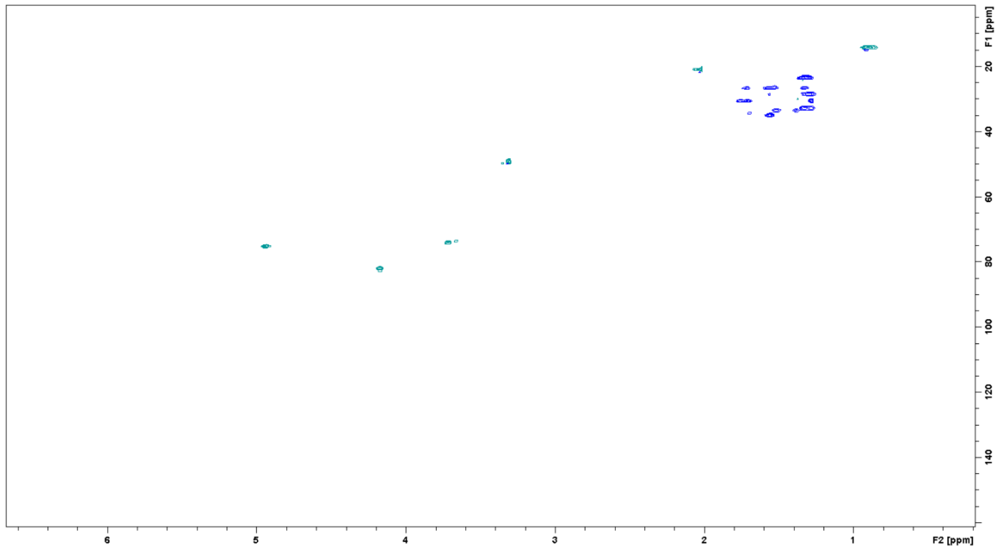

Figure S36. HR-ESIMS spectrum of compound **9b**

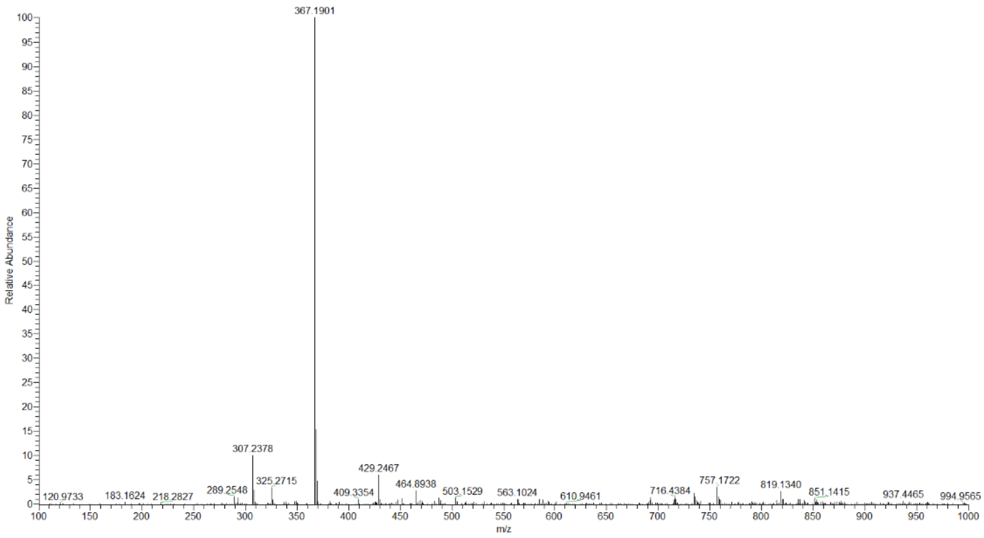

Table S1. Effects of compounds **2**, **3**, **4a**, **4b**, and **8a-9b** on human AR

Compound	% residual activity[a]
**2**	103 ± 4 (71 μM)
**3**	82 ± 3 (71 μM)
**4a**	97 ± 4 (43 μM)
**4b**	98 ± 3 (43 μM)
**8a**	87 ± 2 (71 μM)
**8b**	74 ± 8 (210 μM)
**9a**	86 ± 2 (107 μM)
**9b**	74 ± 6 (210 μM)

The residual activity is reported as the mean ± SD of at least three independent measurements. [a]The maximal concentration of the compound used to evaluate residual activity is reported in parenthesis.
